# Kinesins regulate the heterogeneity in centrosome clustering after whole-genome duplication

**DOI:** 10.26508/lsa.202402670

**Published:** 2024-07-29

**Authors:** Thomas TY Lau, Hoi Tang Ma, Randy YC Poon

**Affiliations:** 1 Division of Life Science, Hong Kong University of Science and Technology , Clear Water Bay, Hong Kong; 2 Department of Pathology, The University of Hong Kong, Pok Fu Lam, Hong Kong; 3 State Key Laboratory of Liver Research, The University of Hong Kong, Pok Fu Lam, Hong Kong; 4 State Key Laboratory of Molecular Neuroscience, Hong Kong University of Science and Technology , Clear Water Bay, Hong Kong

## Abstract

After whole-genome duplication (WGD), tetraploid cells can undergo multipolar mitosis or pseudo-bipolar mitosis with clustered centrosomes. Kinesins play a crucial role in regulating spindle formation. However, the contribution of kinesin expression levels to the heterogeneity in centrosome clustering observed across different cell lines after WGD remains unclear. We identified two subsets of cell lines: “BP” cells efficiently cluster extra centrosomes for pseudo-bipolar mitosis, and “MP” cells primarily undergo multipolar mitosis after WGD. Diploid MP cells contained higher levels of KIF11 and KIF15 compared with BP cells and showed reduced sensitivity to centrosome clustering induced by KIF11 inhibitors. Moreover, partial inhibition of KIF11 or depletion of KIF15 converted MP cells from multipolar to bipolar mitosis after WGD. Multipolar spindle formation involved microtubules but was independent of kinetochore–microtubule attachment. Silencing KIFC1, but not KIFC3, promoted multipolar mitosis in BP cells, indicating the involvement of specific kinesin-14 family members in counteracting the forces from KIF11/KIF15 after WGD. These findings highlight the collective role of KIF11, KIF15, and KIFC1 in determining the polarity of the mitotic spindle after WGD.

## Abstract (precis)

Kinesin levels determine mitotic spindle polarity after whole-genome duplication.

## Introduction

Restricting genome duplication to once per cell cycle is crucial for maintaining genome stability. Whole-genome duplication (WGD) serves as a striking example of genome instability, wherein the entire genome is doubled to create a tetraploid (Lau & Poon, 2023). WGD is one of the most common genomic abnormalities in cancer, with nearly 30% of cancer patients exhibiting tumors that have undergone WGD (Dewhurst et al, 2014; Bielski et al, 2018; Pienta et al, 2022).

Centrosome duplication is a tightly regulated process during the cell cycle, ensuring that centrosomes are duplicated only once per cell cycle (Agircan et al, 2014). In most human cells, the presence of extra centrosomes can result in multipolar mitosis. However, a specific subset of cells has developed mechanisms to either silence or coalesce excess centrosomes, allowing for bipolar mitosis (Sabat-Pośpiech et al, 2019). The ability to cluster extra centrosomes is a crucial event that influences genome stability (Lau & Poon, 2023).

An intriguing question arises regarding why some cell lines can cluster extra centrosomes after WGD, whereas others primarily undergo multipolar mitosis. Seminal work by Quintyne et al demonstrated that supernumerary centrosomes can cluster together, forming pseudo-bipolar spindles (Quintyne et al, 2005). Subsequent genome-wide RNAi screens in *Drosophila* and mammalian models have identified several microtubule-associated motor proteins, including members of the kinesin family such as KIFC1 (HSET), KIF2C, KIF23 (MKLP1), and KIF10, as potential suppressors of multipolar spindle formation (Goshima et al, 2007; Kwon et al, 2008; Leber et al, 2010).

Mitotic kinesins play crucial roles in various mitotic processes, including chromosome alignment and segregation, spindle assembly, and cytokinesis (Cross & McAinsh, 2014). Among the mitotic kinesins, the plus-end–directed KIF11 (also known as kinesin-5 or Eg5) plays a key role in initiating bipolar spindle formation by cross-linking and sliding antiparallel microtubules, exerting outward forces that separate centrosomes (Kapitein et al, 2005). Accordingly, inhibition of KIF11 results in the formation of monopolar spindles (Kapoor et al, 2000; Tao et al, 2005). The action of KIF11 is counteracted by inward forces generated by the minus-end–directed dynein–dynactin, which pulls centrosomes together. Regulators including LIS1 (which enhances dynein’s ATPase activity) and CLIP-170 (a plus-end–tracking protein) work in concert with dynein to generate poleward forces that bring centrosomes closer (Tanenbaum et al, 2008). Other plus-end– and minus-end–directed mitotic kinesins also facilitate and counteract KIF11-mediated centrosome separation, respectively (Mountain et al, 1999; van Heesbeen et al, 2014). Together, these motor proteins contribute to the precise positioning of centrosomes within bipolar spindles. Although KIF11 was not initially identified in genome-wide screens for genes involved in centrosome clustering, subsequent studies revealed its critical role in controlling spindle polarity in tetraploid HeLa and HCT116 cells (Shu et al, 2019).

Given the plethora of candidate genes that can influence centrosome clustering identified from whole-genome screens (Goshima et al, 2007; Kwon et al, 2008; Leber et al, 2010), we aimed to investigate the extent to which the expression of plus-end– and minus-end–directed mitotic kinesins can account for the heterogeneity in centrosome clustering after WGD in different cell lines. Our study revealed that manipulating the expression levels or activities of antagonistic mitotic kinesins, including KIF11, KIF15, and KIFC1, which by themselves do not significantly affect normal mitosis in diploids, could effectively dictate spindle polarity after WGD.

## Results

### Substantial inter-cell line variations in centrosome clustering after WGD

**Figure 1.**
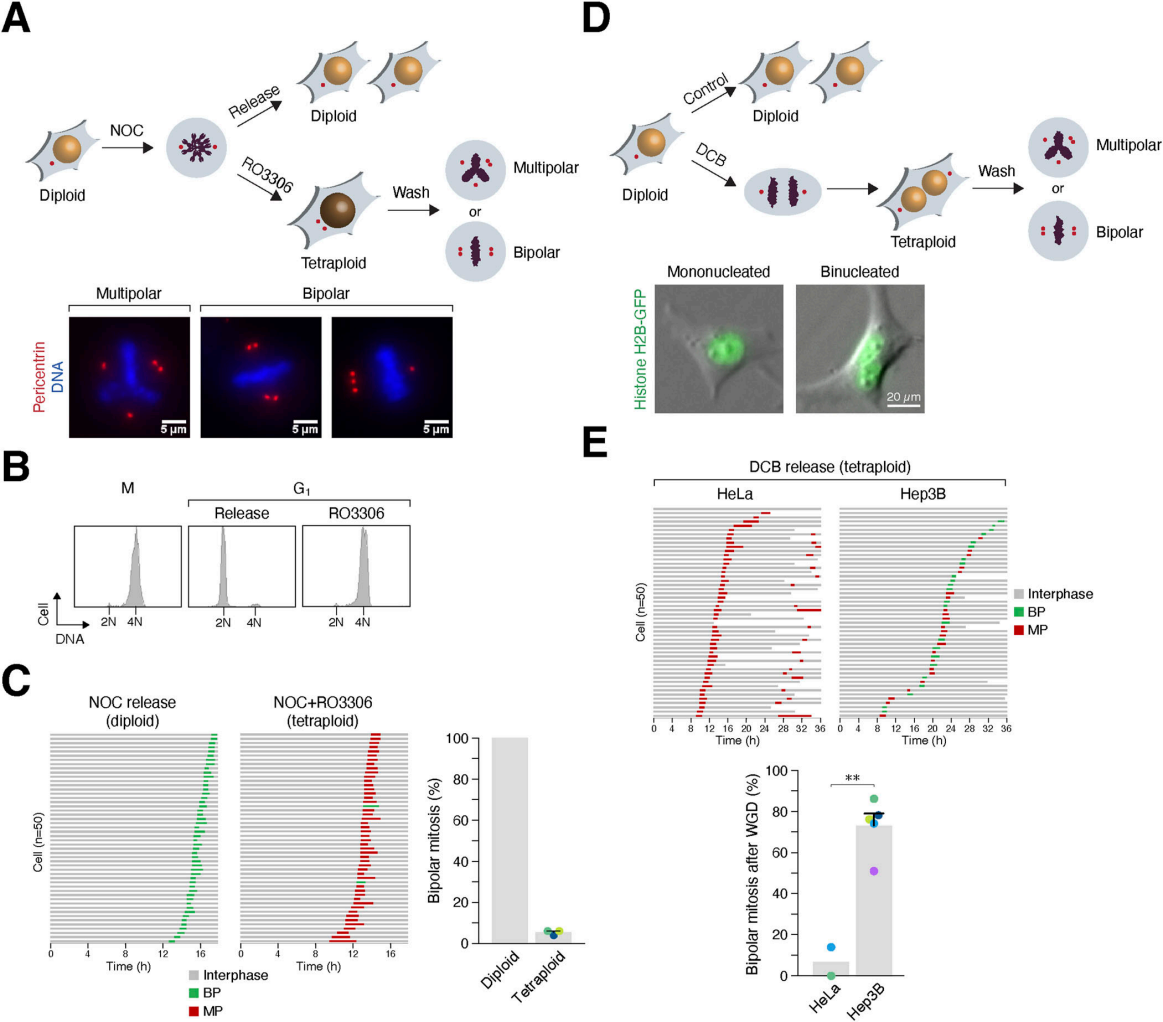
Induction of whole-genome duplication (WGD) by mitotic slippage and cytokinesis failure. **(A)** Generation of tetraploids by inducing mitotic slippage. Cells were first synchronized using a double thymidine procedure and released into nocodazole (NOC)-containing medium. Mitotic cells were isolated through mechanical shake-off and either released into diploid G_1_ by washing out NOC or treated with RO3306 to induce mitotic slippage. After 2 h, chemical inhibitors and unattached cells were removed by washing. The types of mitotic spindles formed during subsequent mitosis were analyzed using live-cell imaging and/or immunofluorescence microscopy. Representative images of tetraploid HeLa cells undergoing mitosis with multipolar or bipolar spindles (centrosome clustering in a 2:2 or 1:3 configuration) are shown. Red: pericentrin; blue: Hoechst 33258. Scale bar = 5 μm. **(B)** Induction of mitotic slippage with RO3306. HeLa cells were blocked in mitosis (M) and then released into either diploid G_1_ or tetraploid G_1_ as described in panel (A). After 3 h, the cells were fixed and analyzed with flow cytometry. **(C)** HeLa cells predominantly undergo multipolar division after WGD. Cells were synchronized in mitosis and then released into either diploid G_1_ or tetraploid G_1_ as described in panel (A). After 2 h, NOC, RO3306, and unattached cells were removed by washing. The fate of individual cells during subsequent mitosis was tracked using time-lapse microscopy. Each horizontal bar represents one cell (n = 50). Key: interphase (gray); mitosis with bipolar division (BP; green); mitosis with multipolar division (MP; red). The graph indicates the mean ± SEM of three independent experiments (for tetraploids). **(D)** Generation of tetraploids by inducing cytokinesis failure. Cells were treated with dihydrocytochalasin B for 18 h to disrupt cleavage furrow assembly. After washing out dihydrocytochalasin B, the fate of binucleated cells was tracked using live-cell imaging. Representative images of mononucleated and binucleated HeLa cells expressing histone H2B-GFP are shown. Scale bar = 20 μm. **(E)** Different cell lines exhibit different extents of bipolar division after WGD. Cytokinesis failure was induced as described in panel (D). The fate of individual binucleated cells during mitosis was tracked using time-lapse microscopy for 36 h. Examples of the analysis from tetraploid HeLa and Hep3B are shown. Key: interphase (gray); mitosis with bipolar division (green); mitosis with multipolar division (red); cell death (truncated bars). The graph indicates the mean of two independent experiments (HeLa) and the mean ± SEM of five independent experiments (Hep3B) (*t* test, **P < 0.01).

**Figure S1.**
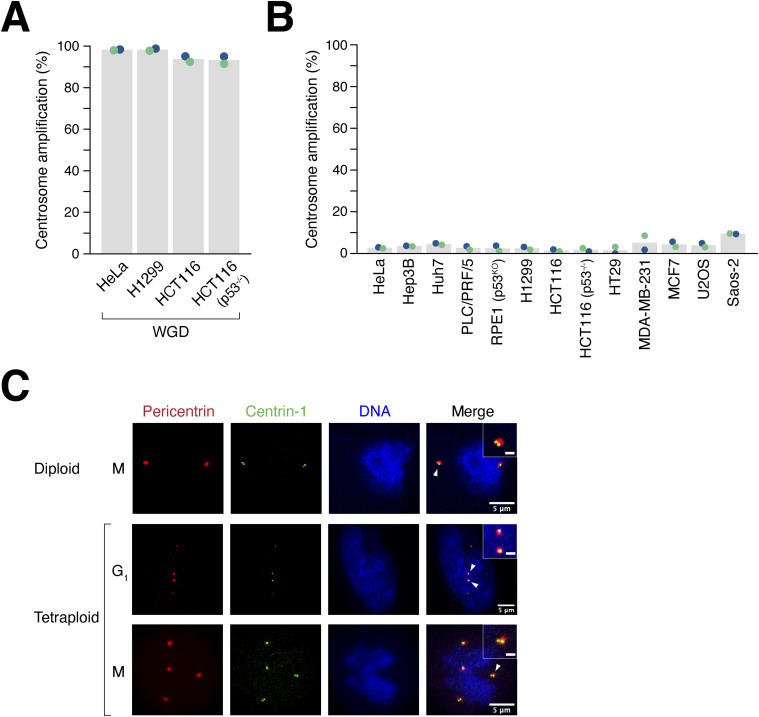
Centrosome amplification after whole-genome duplication (WGD) is not caused by centriole disengagement. **(A)** Induction of centrosome amplification through WGD. WGD was induced through mitotic slippage in the indicated cell lines (Fig 1A). Upon entry into mitosis (∼15 h), the cells were fixed and stained with antibodies against pericentrin and Hoechst 33258 to visualize the centrosomes and DNA, respectively. Cells displaying more than two centrosomes were classified as having an amplified number of centrosomes. The graph represents the percentages of centrosome amplification from two independent experiments (n ≥ 35 for each experiment). **(B)** Centrosome amplification is infrequent before WGD. The indicated diploid cell lines were synchronized using RO3306 (10 μM) for 15 h and then released into mitosis. Cells were then fixed and stained with antibodies against pericentrin and Hoechst 33258 to visualize the centrosomes and DNA, respectively. The graph represents the percentages of centrosome amplification from two independent experiments (n ≥ 52 for each experiment). **(C)** Supernumerary centrosomes are structurally intact. WGD was induced through mitotic slippage (Fig 1A). Cells were fixed and immunostained with antibodies against pericentrin, centrin-1, and Hoechst 33258 to visualize the centrosomes, centrioles, and DNA, respectively. Representative images of HeLa cells before WGD (diploid M), 3 h (tetraploid G_1_), and 15 h after WGD (tetraploid M) are shown. Red: pericentrin; green: centrin-1; blue: Hoechst 33258. Scale bar = 5 μm. Arrows indicate the enlarged centrosomes shown in the insets (scale bar = 1 μm).

Two approaches were used to generate tetraploids in this study. The first method involved synchronizing cells in mitosis using thymidine followed by nocodazole (NOC), followed by treatment with the CDK1 inhibitor RO3306 to trigger mitotic slippage into a tetraploid G_1_ state. The cells were fixed during the following mitosis for analysis using immunofluorescence microscopy. Mitosis was classified as either multipolar (with more than two spindle poles) or bipolar (two spindle poles with centrosome clustering in a 1:3 or 2:2 configuration) (Fig 1A). Alternatively, live-cell imaging was performed to assess mitotic spindle polarity in individual cells. DNA was visualized by either labeling nuclei with a live-cell DNA dye or by expressing histone H2B-GFP/Clover in the cells. Flow cytometry analysis verified the tetraploid DNA content in cells treated with RO3306 (Fig 1B). For cell lines that could be effectively synchronized with this method, >90% of cells contained amplified centrosomes after WGD (Fig S1A). Notably, before WGD, the cell lines used in this study contained <10% of cells with amplified centrosomes (Fig S1B). Centrosomes remained structurally intact after WGD, as centriole duplication occurred normally during the S phase and did not precociously disengage during mitosis (Fig S1C). In the case of HeLa cells, WGD induced by mitotic slippage resulted in almost exclusively multipolar mitosis (Fig 1C).

For cell lines that could not be readily synchronized in mitosis using NOC, a second method using dihydrocytochalasin B (DCB) to abolish cytokinesis was used to generate binucleated tetraploids. The fate of individual binucleated cells was tracked into the subsequent mitosis using live-cell imaging (Fig 1D). Examples of cell lines displaying a high proportion of multipolar mitosis (HeLa) or bipolar mitosis (Hep3B) after cytokinesis failure are shown in Fig 1E.

**Figure 2.**
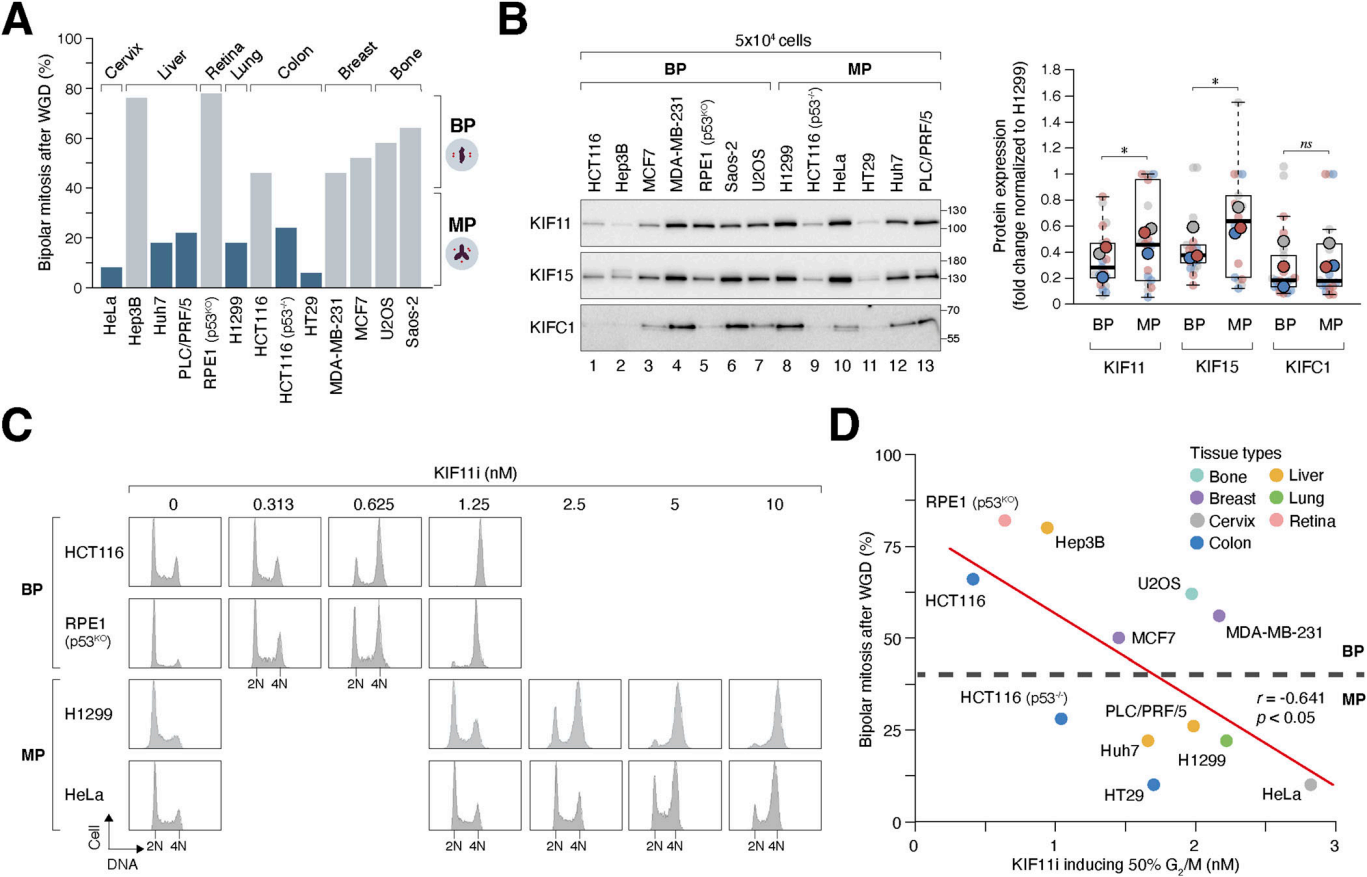
Variations in the frequency of centrosome clustering after whole-genome duplication (WGD) in different cell lines. **(A)** Frequencies of bipolar mitosis after WGD vary among different cell lines. Tetraploidization was induced in the indicated cell lines through either mitotic slippage or cytokinesis failure as described in Fig 1. The percentage of tetraploids undergoing bipolar mitosis was analyzed using live-cell imaging (n = 50). Tissue origins of the cell lines are indicated. Note that as hTERT-immortalized RPE1 cells were arrested in interphase after dihydrocytochalasin B treatment (Ganem et al, 2014), a clone in which the p53 genes were ablated with CRISPR-Cas9 was used. Cell lines are classified as those undergoing >40% bipolar mitosis (“BP”) or >60% multipolar mitosis (“MP”) after WGD. **(B)** Cells forming bipolar spindles after WGD contain relatively low expression of KIF11 and KIF15. Lysates from 5 × 10^4^ cells isolated from the indicated cell lines were analyzed with immunoblotting. The band intensities of KIF11, KIF15, and KIFC1 in different cell lines were quantified and normalized to that in H1299 cells (which contain the highest level of KIF11). Box-and-whisker plots represent the expression of KIF11, KIF15, and KIFC1 in BP (n = 7) and MP (n = 6) cell lines from three independent experiments (the means from each experiment are shown; *t* test, *P < 0.05; ns *P* > 0.05). **(C)** Varying sensitivity to KIF11i among different cell lines. Cells were incubated with buffer or serially diluted KIF11i (SB743921) for 24 h. The cells were then fixed and analyzed using flow cytometry. Selected cell lines from “BP” and “MP” groups are shown. Refer to Fig S2B for data from other cell lines. **(D)** Correlation between KIF11i sensitivity in diploids and frequency of bipolar mitosis after WGD. The indicated cell lines were incubated with different concentrations of KIF11i for 24 h and analyzed with flow cytometry. The EC_50_ values of KIF11i for inducing G_2_/M arrest were determined. Percentage of cells undergoing bipolar mitosis after WGD was measured using live-cell imaging as described in panel (A) (linear regression, *P < 0.05).

To determine the proportion of cells undergoing bipolar or multipolar mitosis after WGD, we performed live-cell imaging to analyze various cell lines from different tissue origins (Fig 2A). Our analysis revealed a significant variability in the ability of tetraploids to undergo mitosis with bipolar spindles. The frequency of bipolar mitosis ranged from ∼10% (HeLa) to as high as 80% (RPE1), highlighting the considerable cell line variations in the ability of tetraploids to cluster extra centrosomes.

### Cell lines with high sensitivity to KIF11 inhibition correlate with the frequency of centrosome clustering after WGD

**Figure S2.**
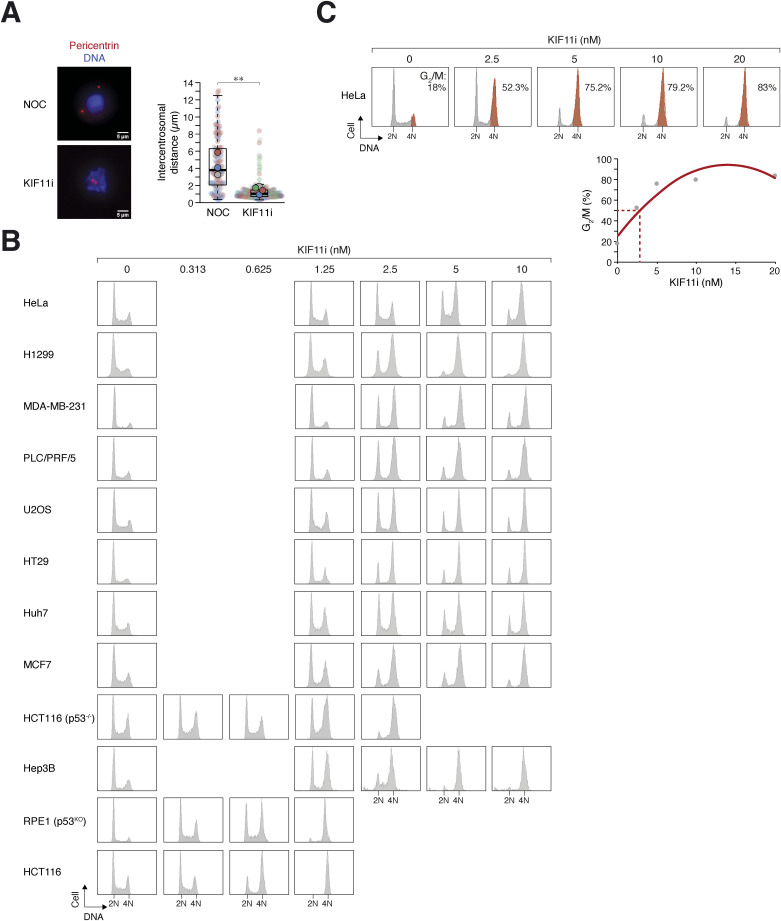
Cell line–specific sensitivity to KIF11i-induced monopolar arrest. **(A)** KIF11 inhibition impedes centrosome separation. HeLa cells were synchronized by double thymidine block and released into nocodazole (NOC)- or KIF11i (50 nM)-containing medium. Upon entry into mitosis (∼9 h), the cells were fixed and stained with antibodies against pericentrin and Hoechst 33258 to visualize the centrosomes and DNA, respectively. Representative images of mitotic cells arrested by NOC and KIF11i are shown. Red: pericentrin; blue: Hoechst 33258. Scale bar = 5 μm. Box-and-whisker plots show the combined data from three (NOC) and four (KIF11i) independent experiments (the means from each experiment are shown; n ≥ 19 for each experiment; *t* test, **P < 0.01). **(B)** Varying sensitivity to KIF11i among different cell lines. The indicated cell lines were incubated with buffer or serially diluted KIF11i for 24 h. The cells were then fixed and analyzed with flow cytometry. Note that selected samples are shown in Fig 2C. **(C)** Estimation of EC_50_ of KIF11i using flow cytometry. Cell lines (HeLa shown as an example) were incubated with different concentrations of KIF11i for 24 h. The cells were then fixed and analyzed with flow cytometry. The percentage of cells with G_2_/M DNA content was quantified (highlighted in red) and plotted against the KIF11i concentration. EC_50_ of KIF11i in a cell line was extrapolated from the concentration required to arrest half of the cells in G_2_/M using a polynomial trendline.

Cell lines were categorized into two groups based on their ability to cluster extra centrosomes after WGD: “BP” cell lines that efficiently clustered extra centrosomes after WGD (>40% of mitosis was bipolar) and “MP” that clustered extra centrosomes ineffectively (Fig 2A). Analysis of the BP group revealed relatively lower expression levels of the plus-end–directed motor KIF11 and KIF15 compared with the MP group (Fig 2B). To investigate whether the lower expression of KIF11 in the MP group correlated with sensitivity to KIF11 inhibition, we employed a specific small chemical KIF11 inhibitor SB743921, which binds to an allosteric pocket formed by helix α2/loop L5/helix α3 and showed greater than 40,000-fold selectivity for KIF11 over other kinesins (Holen et al, 2011; Talapatra et al, 2013). Treatment with high concentrations of SB743921 (KIF11i herein) promoted monopolar spindle formation and mitotic arrest, as shown by flow cytometry analysis (Fig 2C), and decreased the intercentrosomal distance (Fig S2A).

We found that different cell lines displayed varying sensitivity to KIF11i (Figs 2C and S2B). MP cell lines, such as HeLa and H1299, were relatively insensitive to KIF11i, requiring higher concentrations to induce G_2_/M arrest. In comparison, BP cell lines, such as HCT116 and RPE1, were more susceptible to KIF11i. The EC_50_ of KIF11i for inducing G_2_/M arrest was estimated using flow cytometry analysis of cell lines treated with serially diluted KIF11i (Fig S2C). A strong correlation was observed between KIF11i sensitivity in diploid cells and the frequency of bipolar mitosis after WGD (Fig 2D), suggesting that KIF11 activity may serve as a key determinant of mitotic spindle polarity after WGD.

### Extra spindle poles increase KIF11–microtubule association and suppress bipolar spindle formation in “MP” cells

**Figure S3.**
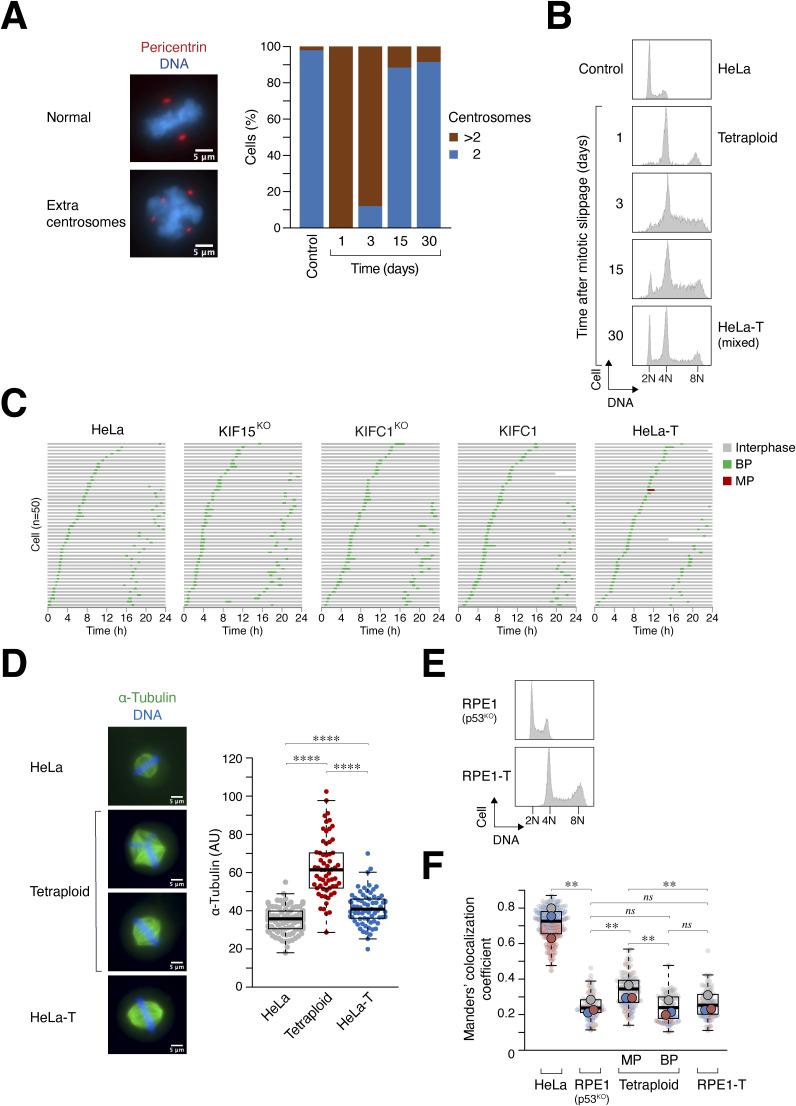
Progressive loss of extra centrosomes after whole-genome duplication (WGD). **(A)** Progressive loss of extra centrosomes after WGD. Tetraploid HeLa cells were generated by mitotic slippage (Fig 1A). The cells were cultured in KIF11i-containing medium for 24 h. Subsequently, cells were continuously subcultured in normal medium and fixed on the indicated days for analysis using immunofluorescence microscopy. The number of centrosomes per cell during mitosis was quantified (n = 50). Representative images of cells containing two or more than two centrosomes during mitosis are shown. Red: pericentrin; blue: Hoechst 33258. Scale bar = 5 μm. **(B)** Portion of WGD cells remain in a near-tetraploid state after prolonged passaging. **(A)** Tetraploid HeLa cells were generated as described in panel (A) and fixed for flow cytometry analysis on the indicated days. The positions of 2N, 4N, and 8N DNA contents are indicated. **(C)** Relatively normal cell proliferation in KIF15 knockout, KIFC1 knockout, and KIFC1-overexpressing HeLa diploids, and stable HeLa tetraploids. The indicated cell lines derived from HeLa cells were analyzed with live-cell imaging (n = 50). Key: interphase (gray); bipolar mitosis (green); multipolar mitosis (red); cell death (truncated bars). **(D)** Extra centrosomes increase microtubule emanation. Synchronized diploid, tetraploid HeLa cells, and HeLa-T cells were generated as described in Fig 1A. Cells were fixed and stained with antibodies against ⍺-tubulin and Hoechst 33258 to visualize the microtubules and DNA, respectively. Representative images of mitotic diploid HeLa, tetraploids (both bipolar and multipolar spindles), and HeLa-T are shown. Green: ⍺-tubulin; blue: Hoechst 33258. Scale bar = 5 μm. Box-and-whisker plots show the average intensity of ⍺-tubulin of individual cells (*n* ≥ 62; Mann–Whitney test, ****P < 0.0001). **(E)** Generation of stable RPE1 tetraploids. WGD was induced in RPE1 (p53^KO^) cells as described in Fig 1A. Clones with a near-tetraploid DNA content and normal centrosome number were isolated (RPE1-T). The DNA contents of RPE1 (p53^KO^) and RPE1-T were analyzed with flow cytometry. **(F)** Multipolar mitosis promotes KIF11 localization on mitotic spindles. Tetraploid RPE1 (p53^KO^) cells were generated through mitotic slippage (Fig 1A). Diploid RPE1 (p53^KO^) and stable tetraploid RPE1-T cells were prepared by releasing nocodazole-arrested mitotic cells into interphase. After 18 h, the cells were fixed and analyzed using immunofluorescence microscopy. Mitotic spindles in tetraploid RPE1 (p53^KO^) were classified as bipolar (BP) or multipolar (MP). Diploid HeLa cells in metaphase were also analyzed (note that the data are the same as in Fig 3D for comparison). The localization of KIF11 to microtubules was quantified using Manders’ colocalization coefficient. Box-and-whisker plots show the combined data from three independent experiments (*n* ≥ 20 for each experiment; the individual means of the three experiments are shown; *t* test, **P < 0.01; ns *P* > 0.05).

**Figure 3.**
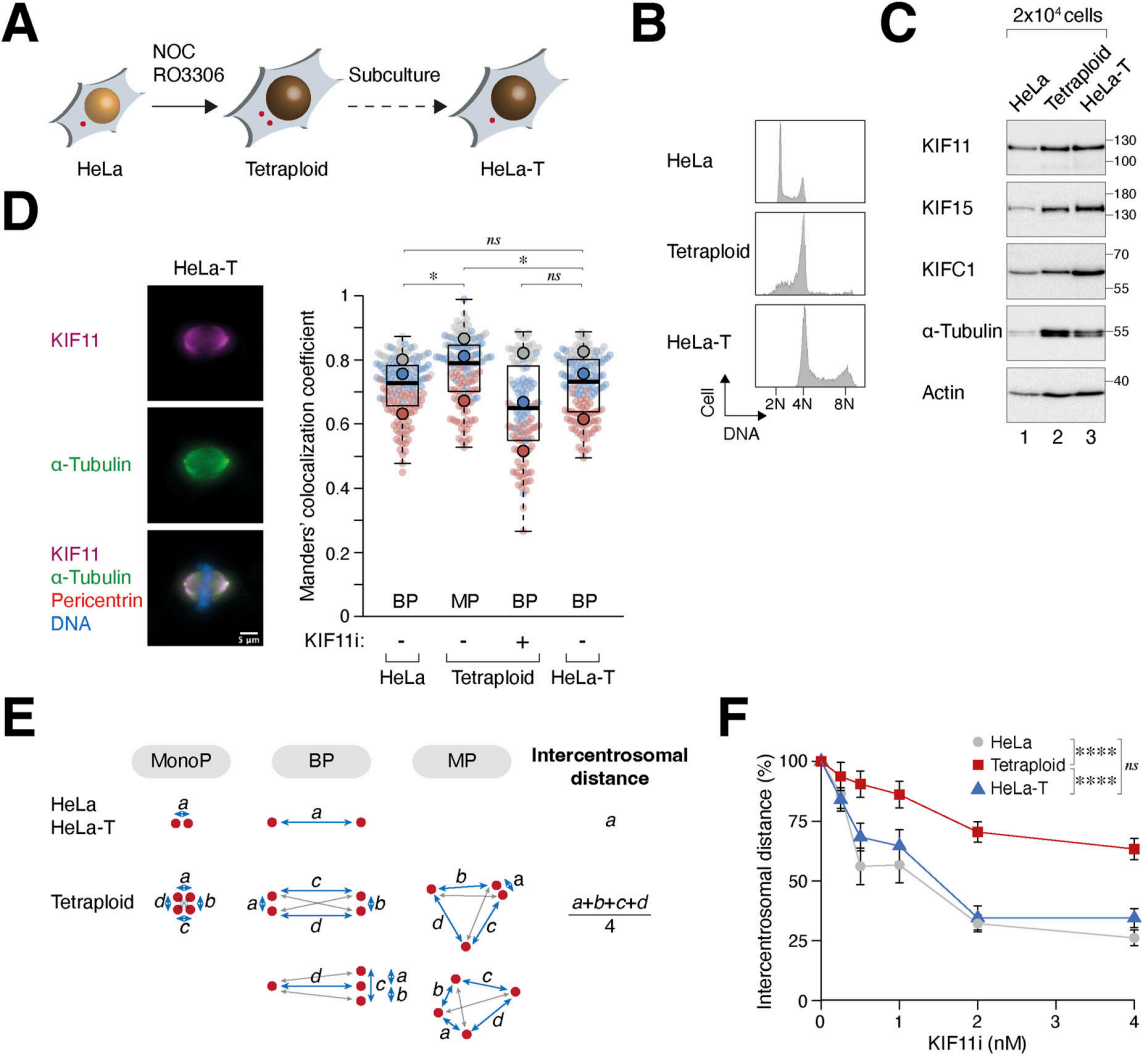
Extra spindle poles increase KIF11–microtubule association. **(A)** Generation of stable tetraploids. Whole-genome duplication (WGD) was induced in HeLa cells as described in Fig 1A. Clones with a near-tetraploid DNA content and the normal number of centrosomes were isolated by limiting dilution (HeLa-T). **(B)** Freshly generated tetraploids and stable HeLa-T contain tetraploid DNA. The DNA contents of the two types of tetraploids (fixed immediately after WGD or after single-colony isolation) and normal HeLa cells were analyzed with flow cytometry. **(C)** Tetraploids contain higher levels of KIF11 and tubulin. The indicated cells were blocked in mitosis with nocodazole for 18 h followed by shake-off. Lysates from 2 × 10^4^ cells per lane were analyzed with immunoblotting. **(D)** Centrosome amplification promotes KIF11 localization on mitotic spindles. WGD was induced in HeLa cells to generate tetraploids (Fig 1A). Diploid HeLa and tetraploid HeLa-T cells were prepared by releasing nocodazole-arrested mitotic cells into interphase. After treatment with either buffer or KIF11i for 15 h, the cells were fixed and analyzed using immunofluorescence microscopy. Representative images of HeLa-T undergoing bipolar mitosis are shown. Red: pericentrin; green: ⍺-tubulin; magenta: KIF11; blue: Hoechst 33258. Scale bar = 5 μm. The localization of KIF11 to microtubules was quantified using Manders’ colocalization coefficient. Box-and-whisker plots show the data from three independent experiments (the individual means of the three experiments are shown; n ≥ 30 for each experiment; *t* test, *P < 0.05; ns *P* > 0.05). Whether the mitosis was predominantly bipolar (BP) or multipolar (MP) in different conditions is indicated. **(E)** Method for estimating centrosome separation in diploids and tetraploids. The intercentrosomal distance in cells with two centrosomes (HeLa and HeLa-T) is defined as the three-dimensional distance between two pericentrin foci. For tetraploids with supernumerary centrosomes, the intercentrosomal distance was calculated by the average of the four shortest three-dimensional intercentrosomal distances. Note that the calculation gives a different value of the intercentrosomal distance between the two types of bipolar spindles in tetraploids (2:2 or 1:3 centrosome clustering). **(F)** Extra centrosomes decrease sensitivity to KIF11 inhibition. The indicated cells were treated with buffer or serially diluted KIF11i for 15 h before being fixed and analyzed using immunofluorescence microscopy. Intercentrosomal distances of individual cells were normalized to their untreated controls (n = 30; mean ± SEM; Dunn–Bonferroni test, ****P < 0.0001; ns *P* > 0.05).

To gain further insights into the relationship between KIF11, WGD, and centrosome number, we generated isogenic tetraploid cells with varying centrosome numbers. After WGD, tetraploid HeLa cells initially contained extra centrosomes that gradually decreased to the normal number upon subsequent passaging (Fig S3A). Notably, a subpopulation maintained a near-tetraploid DNA content (Fig S3B). We isolated single-cell–derived clones from these cells, referred to as HeLa-T (Fig 3A). Flow cytometry analysis verified that both freshly generated tetraploids and HeLa-T cells contained a near-tetraploid DNA content (Fig 3B). Furthermore, HeLa-T cells proliferated normally with bipolar mitosis (Fig S3C).

Because of their larger cell volume compared with diploids, both freshly generated tetraploid HeLa and HeLa-T cells contained higher protein levels when the same number of cells was analyzed (Fig 3C; see actin expression). However, immunoblotting (Fig 3C) and immunostaining of metaphase cells (Fig S3D) revealed higher levels of tubulin in freshly generated tetraploid cells compared with diploid HeLa or HeLa-T. In agreement with previous studies (Godinho et al, 2014), these results suggest that the presence of extra centrosomes leads to increased nucleation of centrosomal microtubules. Interestingly, a higher colocalization of KIF11 with microtubules was observed in freshly generated tetraploids undergoing multipolar mitosis compared with stable bipolar tetraploid HeLa-T (Fig 3D).

Similarly, we examined the “BP” cell line RPE1, which underwent bipolar mitosis in up to 80% of cells after WGD (Fig 2A). Stable tetraploid RPE1 cells that contained two centrosomes during mitosis were isolated (RPE1-T; Fig S3E). Interestingly, we observed a relatively lower colocalization index between KIF11 and microtubules in RPE1 compared with HeLa cells (Fig S3F). Nevertheless, there was an increase in KIF11–microtubule colocalization in freshly generated tetraploid RPE1 cells, but not in stable tetraploid RPE1-T cells. As in HeLa cells, the colocalization of KIF11 with microtubules was stronger in tetraploid RPE1 cells undergoing multipolar mitosis compared with those undergoing bipolar mitosis in stable tetraploid RPE1-T cells. These results suggest that the increase in KIF11 association with microtubules depends on the presence of extra spindle poles and is not solely influenced by tetraploidization.

By estimating the degree of centrosome separation (Fig 3E), we found that KIF11i promoted similar centrosome clustering in both diploid HeLa and stable tetraploid HeLa-T cells. This indicates that the presence of extra chromosomes does not significantly impede KIF11i-induced centrosome clustering. In contrast, freshly generated tetraploids containing extra centrosomes were relatively less sensitive to KIF11i in inducing centrosome clustering (Fig 3F).

Collectively, the correlation between the increase in KIF11 on mitotic spindles and the formation of multipolar spindles after WGD suggests that KIF11 likely plays a crucial role in determining spindle polarity after WGD.

### KIF11 suppresses supernumerary centrosome clustering after WGD

**Figure 4.**
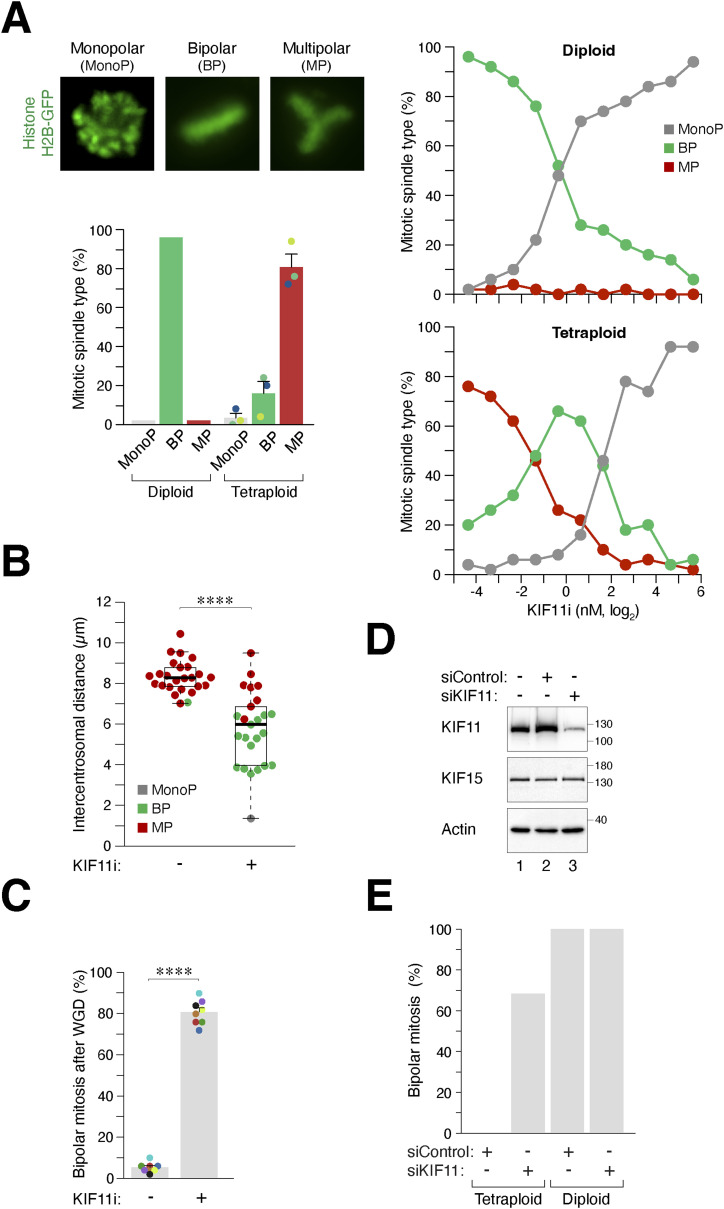
KIF11 suppresses bipolar spindle formation after whole-genome duplication (WGD). **(A)** KIF11 activity determines the number of spindle poles in both diploids and tetraploids. Synchronized diploids and tetraploids were generated using HeLa cells expressing histone H2B-GFP (Fig 1A). The cells were treated with serial dilutions of KIF11i for 15 h. The percentages of cells undergoing monopolar, bipolar, and multipolar mitosis were determined using immunofluorescence microscopy (n = 50). Representative examples and the percentages of different mitotic spindle polarity in untreated diploids and tetraploids are shown on the left (mean ± SEM from three independent experiments for tetraploids). **(B)** Inhibition of KIF11 promotes centrosome clustering after WGD. WGD was induced in synchronized HeLa cells (Fig 1A). The cells were left untreated or treated with KIF11i (1 nM). Upon entry into mitosis (∼15 h), the cells were fixed and stained with antibodies against pericentrin and Hoechst 33258 to visualize the centrosomes and DNA, respectively. Intercentrosomal distances were measured according to the method described in Fig 3E (box-and-whisker plots; n = 25; Mann–Whitney test, ****P < 0.0001). **(C)** Partial inhibition of KIF11 switches multipolar mitosis to bipolar mitosis after WGD. Tetraploid HeLa cells generated through mitotic slippage (Fig 1A) were cultured in the presence or absence of KIF11i (SB743921, 1 nM). The mitotic cell fate was analyzed using time-lapse microscopy (n = 50 for each experiment). The percentage of cells undergoing bipolar mitosis after WGD was quantified from eight independent experiments (mean ± SEM; *t* test, ****P < 0.0001). **(D)** siRNA-mediated knockdown of KIF11. Diploid HeLa cells were transiently transfected with a control or KIF11 siRNA. A relatively low concentration of siRNA was used to partially down-regulate KIF11 expression. After 24 h, cells were harvested and analyzed with immunoblotting. **(E)** Partial knockdown of KIF11 alters the mitotic cell fate in tetraploids but not in diploids. Diploid HeLa cells expressing histone H2B-GFP were transfected with a control or KIF11 siRNA. WGD was induced through mitotic slippage (Fig 1A). The mitotic cell fate was then analyzed using live-cell imaging for 24 h (n = 50). The raw live-cell imaging data can be found in Fig S4F.

To determine whether the change in KIF11 expression is a causal factor or a consequence of the presence of extra spindle poles in “MP” cells after WGD, we treated diploid or freshly generated tetraploid HeLa cells with serially diluted KIF11i (Fig 4A). As expected, both diploids and tetraploids were arrested in mitosis with monopolar spindles at high concentrations of KIF11i. Notably, partial inhibition of KIF11 shifted the predominantly multipolar spindles in tetraploids to bipolar spindles. This was accompanied by a decrease in the intercentrosomal distance (Fig 4B).

**Figure S4.**
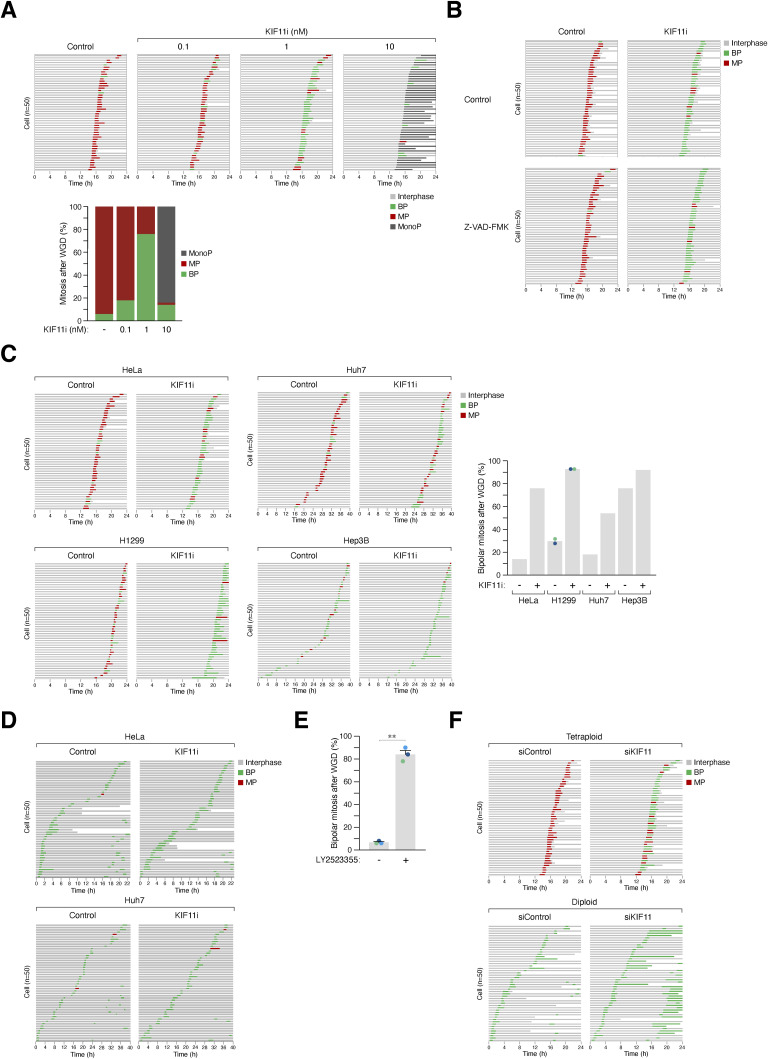
KIF11 suppresses bipolar division after whole-genome duplication (WGD). **(A)** Partial inhibition of KIF11 after WGD switches multipolar mitosis to bipolar mitosis. WGD was induced in synchronized HeLa cells through mitotic slippage (Fig 1A). The cells were then cultured in the absence or presence of the indicated concentrations of KIF11i. The mitotic cell fate of individual cells was then analyzed using time-lapse microscopy (n = 50). Key: interphase (gray); bipolar mitosis (green); multipolar mitosis (red); monopolar arrest (dark gray); cell death (truncated bars). The percentages of different mitotic cell fates were quantified. **(B)** Mitotic cell fate in tetraploids is independent of apoptosis. WGD was induced in synchronized HeLa cells through mitotic slippage (Fig 1A). The cells were cultured in the absence or presence of KIF11i and/or pan-caspase inhibitor (Z-VAD-FMK). The mitotic cell fate of individual cells was then analyzed using time-lapse microscopy (n = 50). Key: interphase (gray); bipolar mitosis (green); multipolar mitosis (red); cell death (truncated bars). **(C)** Bipolar mitosis promoted by KIF11 inhibition after WGD is not specific to a particular cell type. WGD was induced in synchronized H1299 cells (expressing histone H2B-Clover) through mitotic slippage (Fig 1A). Cytokinesis failure was triggered in HeLa, Huh7, and Hep3B (expressing histone H2B-GFP) using dihydrocytochalasin B (Fig 1C). The cells were then cultured in the absence or presence of KIF11i (0.5 nM H1299 and 1 nM for the others). The mitotic cell fate of tetraploid cells was analyzed using live-cell imaging (n = 50). Key: interphase (gray); bipolar mitosis (green); multipolar mitosis (red); cell death (truncated bars). The percentage of bipolar mitosis was quantified (data were from two independent experiments [H1299] or one experiment [other cell lines]). **(D)** Diploid cells undergo bipolar mitosis in the presence of KIF11i. Cytokinesis failure was induced in HeLa and Huh7 (expressing histone H2B-GFP) using dihydrocytochalasin B (Fig 1C). The cells were then cultured in the absence or presence of KIF11i (1 nM). The cell fate of diploid cells (containing single nucleus) was tracked using live-cell imaging (n = 50). Key: interphase (gray); bipolar mitosis (green); multipolar mitosis (red); cell death (truncated bars). **(E)** Partial inhibition of KIF11 promotes bipolar mitosis after WGD. Tetraploid HeLa cells generated through mitotic slippage (Fig 1A) were cultured in the presence or absence of KIF11i (LY2523355, 2 nM). The mitotic cell fate was analyzed using time-lapse microscopy (n = 50 for each experiment). The graph indicates the percentages of bipolar mitosis after WGD from three independent experiments (mean ± SEM; *t* test, **P < 0.01). **(F)** Partial knockdown of KIF11 alters the mitotic cell fate in tetraploids but not in diploids. HeLa cells expressing histone H2B-GFP were transfected with a control or KIF11 siRNA. WGD was induced through mitotic slippage (Fig 1A). The mitotic cell fate of the tetraploids and diploids (asynchronous) was then analyzed using live-cell imaging for 24 h (n = 50). Key: interphase (gray); bipolar mitosis (green); multipolar mitosis (red); cell death (truncated bars).

To validate the results obtained from static microscopic images, we performed live-cell imaging and found that treatment with 1 nM of KIF11i converted the predominantly multipolar mitosis after WGD into bipolar mitosis in HeLa cells (Fig 4C). As expected, further increasing the concentration of KIF11i led to the formation of monopolar spindles (Fig S4A). Caspases have been implicated in preventing centrosome amplification (Fava et al, 2017). However, we found that the presence of the pan-caspase inhibitor Z-VAD-FMK did not affect the promotion of bipolar mitosis by KIF11i, suggesting that this effect is independent of apoptosis associated with WGD (Fig S4B).

The promotion of bipolar mitosis by KIF11i was not limited to HeLa cells, as bipolar mitosis was enhanced by KIF11i in cell lines originating from different tissues and exhibiting different intrinsic frequencies of bipolar mitosis after WGD. Treatment with KIF11i increased the percentage of binucleated cells undergoing bipolar mitosis in HeLa, H1299, Huh7, and Hep3B cells (Fig S4C). In contrast, control single-nucleus (diploid) cells treated with DCB underwent bipolar mitosis regardless of KIF11i treatment (Fig S4D).

To ensure the specificity of the effect of KIF11i on spindle polarity after WGD, we treated cells with another KIF11 inhibitor, LY2523355 (Ye et al, 2015), and showed that it also promoted bipolar mitosis after WGD (Fig S4E). Furthermore, down-regulation of KIF11 expression using a relatively low concentration of siRNA (Fig 4D) did not induce monopolar arrest in diploids but was sufficient to promote bipolar mitosis in tetraploids (Figs 4E and S4F).

Taken together, these data indicate that partial inhibition or down-regulation of KIF11 facilitates the conversion of cells from the “MP” group, characterized by multipolar mitosis, to bipolar mitosis after WGD.

### Combined action of KIF15 and KIF11 suppresses bipolar mitosis after WGD

**Figure 5.**
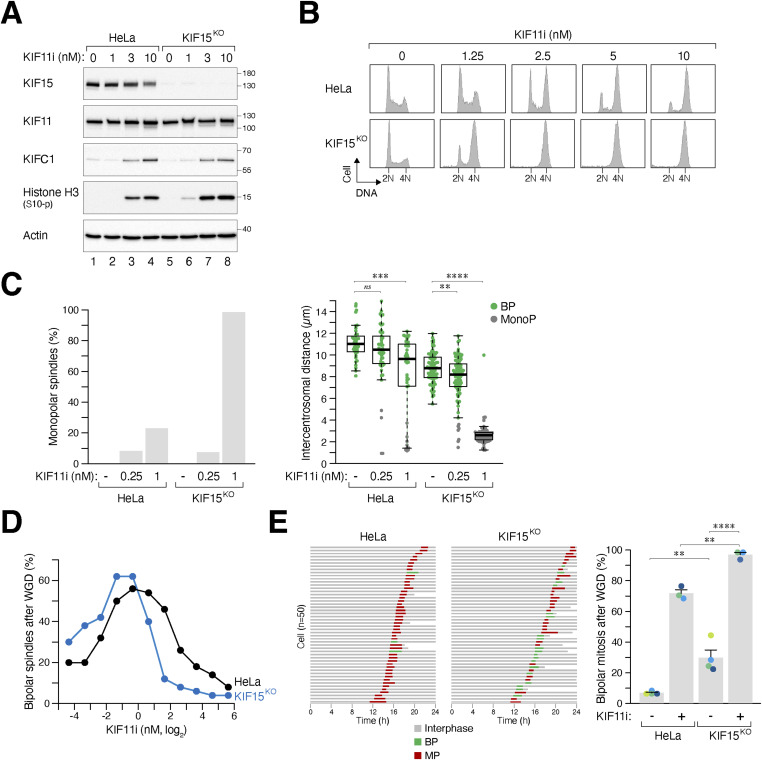
Collaborative role of KIF15 and KIF11 in suppressing bipolar mitosis after whole-genome duplication (WGD). **(A)** KIF15 knockout increases sensitivity to KIF11i-induced mitotic arrest in diploids. The *KIF15* gene was disrupted in diploid HeLa cells using CRISPR-Cas9. Diploid HeLa and KIF15^KO^ cells were treated with different concentrations of KIF11i for 18 h. Lysates were prepared and analyzed with immunoblotting. **(B)** Silencing of KIF15 increases sensitivity to KIF11i-induced mitotic arrest in diploids. Diploid HeLa and KIF15^KO^ cells were treated with different concentrations of KIF11i for 18 h and analyzed using flow cytometry. **(C)** Silencing of KIF15 promotes the KIF11i-induced monopolar spindle formation in diploids. Diploid HeLa and KIF15^KO^ cells were synchronized with double thymidine block and released into medium with or without the indicated concentrations of KIF11i. Once cells entered mitosis (∼9 h), they were fixed and stained with Hoechst 33258 to visualize the DNA. The percentage of mitotic cells displaying monopolar spindles was quantified (left panel). Intercentrosomal distances were measured by immunostaining for pericentrin (right panel) (box-and-whisker plots; *n* ≥ 40; Mann–Whitney test, ****P < 0.0001; ***P < 0.001; **P < 0.01; ns *P* > 0.05). **(D)** Combined action of KIF15 and KIF11 in suppressing bipolar mitosis in tetraploids. WGD was induced in synchronized HeLa and KIF15^KO^ cells (Fig 1A). The cells were incubated with serial dilutions of KIF11i. The percentage of cells forming bipolar spindles was determined with immunofluorescence microscopy (n = 50). **(E)** Silencing of KIF15 promotes bipolar division in tetraploids. WGD was induced in synchronized HeLa and KIF15^KO^ cells (Fig 1A). The mitotic cell fate of individual cells, treated with either buffer or KIF11i (0.25 nM), was then tracked with time-lapse microscopy (n = 50). Key: interphase (gray); bipolar mitosis (green); multipolar mitosis (red); cell death (truncated bars). The percentage of cells undergoing bipolar mitosis after WGD was quantified from three independent experiments (mean ± SEM; *t* test, ****P < 0.0001; **P < 0.01).

We observed a decrease in KIF15 expression in cells from the “BP” group compared with the “MP” group (Fig 2B). In diploids, KIF11 and KIF15 are functionally redundant in generating outward pushing forces that separate spindle poles (Tanenbaum et al, 2009). To investigate the role of KIF15 after WGD, we used CRISPR-Cas9 to disrupt the *KIF15* gene. We found that knockout of KIF15 in HeLa cells did not affect mitosis or cell cycle progression in diploids (Fig S3C). Consistent with the overlapping function of KIF15 with KIF11, KIF15^KO^ cells showed increased sensitivity to KIF11i, as indicated by the stronger accumulation of histone H3^Ser10^ phosphorylation (Fig 5A), G_2_/M population (Fig 5B), and the formation of monopolar spindles (Fig 5C).

**Figure S5.**
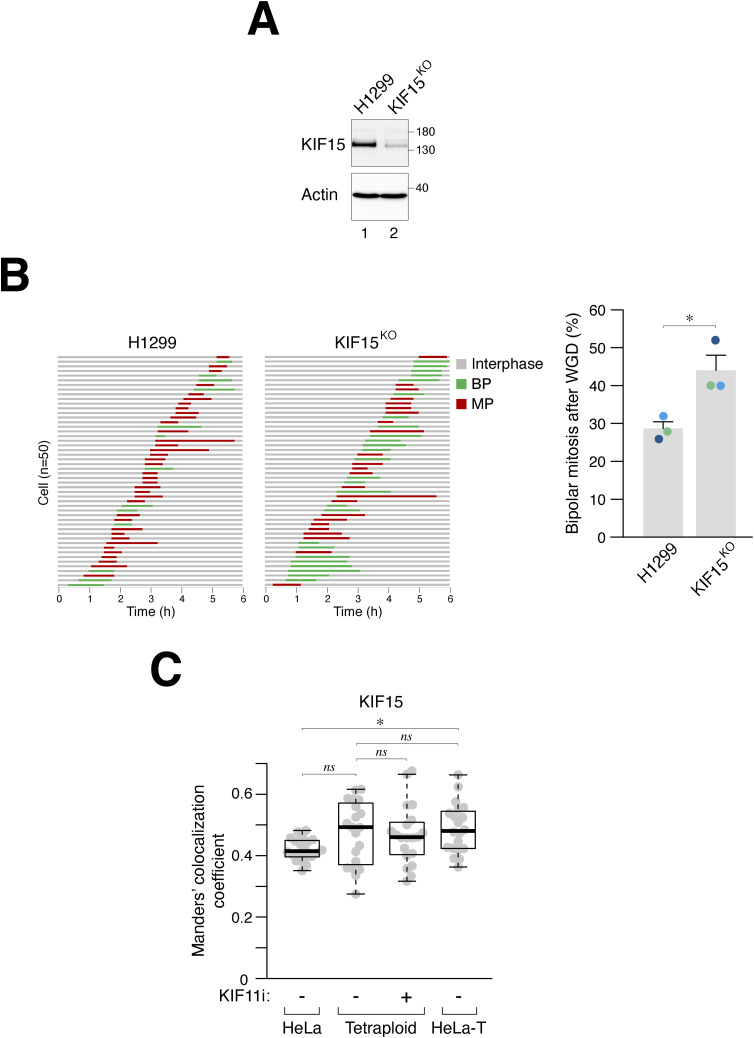
Collaborative role of KIF15 and KIF11 in suppressing bipolar mitosis in tetraploids. **(A)** Silencing of KIF15 in H1299 cells. The *KIF15* gene was disrupted in H1299 cells using CRISPR-Cas9. Blasticidin selection was applied for 48 h to obtain a mixed population of KIF15^KO^ H1299 cells. Lysates were then prepared and analyzed with immunoblotting. **(B)** Silencing of KIF15 in H1299 cells promotes bipolar mitosis after whole-genome duplication (WGD). WGD was induced in synchronized normal and KIF15^KO^ H1299 cells (Fig 1A). The mitotic cell fate was monitored using time-lapse microscopy (n = 50). Key: interphase (gray); bipolar mitosis (green); multipolar mitosis (red). The graph indicates the percentages of bipolar mitosis after WGD from three independent experiments (mean ± SEM; *t* test, *P < 0.05). **(C)** Centrosome amplification does not increase KIF15 localization on mitotic spindles. WGD was induced in HeLa cells to generate tetraploids (Fig 1A). Diploid HeLa and tetraploid HeLa-T cells were prepared by releasing nocodazole-arrested mitotic cells into interphase. After treatment with either buffer or KIF11i for 15 h, the cells were fixed and analyzed using immunofluorescence microscopy. The localization of KIF15 to microtubules was quantified using Manders’ colocalization coefficient (n = 20; Mann–Whitney test, *P < 0.05; ns *P* > 0.05).

After WGD, knockout of KIF15 alone slightly increased the percentage of bipolar spindles (Fig 5E). However, the loss of KIF15 further enhanced the KIF11i-induced bipolar mitosis, as revealed by both immunofluorescence microscopy of fixed samples (Fig 5D) and live-cell imaging (Fig 5E). Similar results were obtained with a mixed population of KIF15^KO^ cells from another “MP” cell line H1299 (Fig S5A and B). Unlike KIF11 (Figs 3D and S3F), the localization of KIF15 on microtubules did not significantly change in tetraploid cells containing extra centrosomes (Fig S5C). Taken together, these data indicate that KIF15 corroborates with KIF11 to suppress bipolar mitosis after WGD.

### Microtubules, but not kinetochore–microtubule attachment, are required for KIF11-dependent inhibition of centrosome clustering

**Figure 6.**
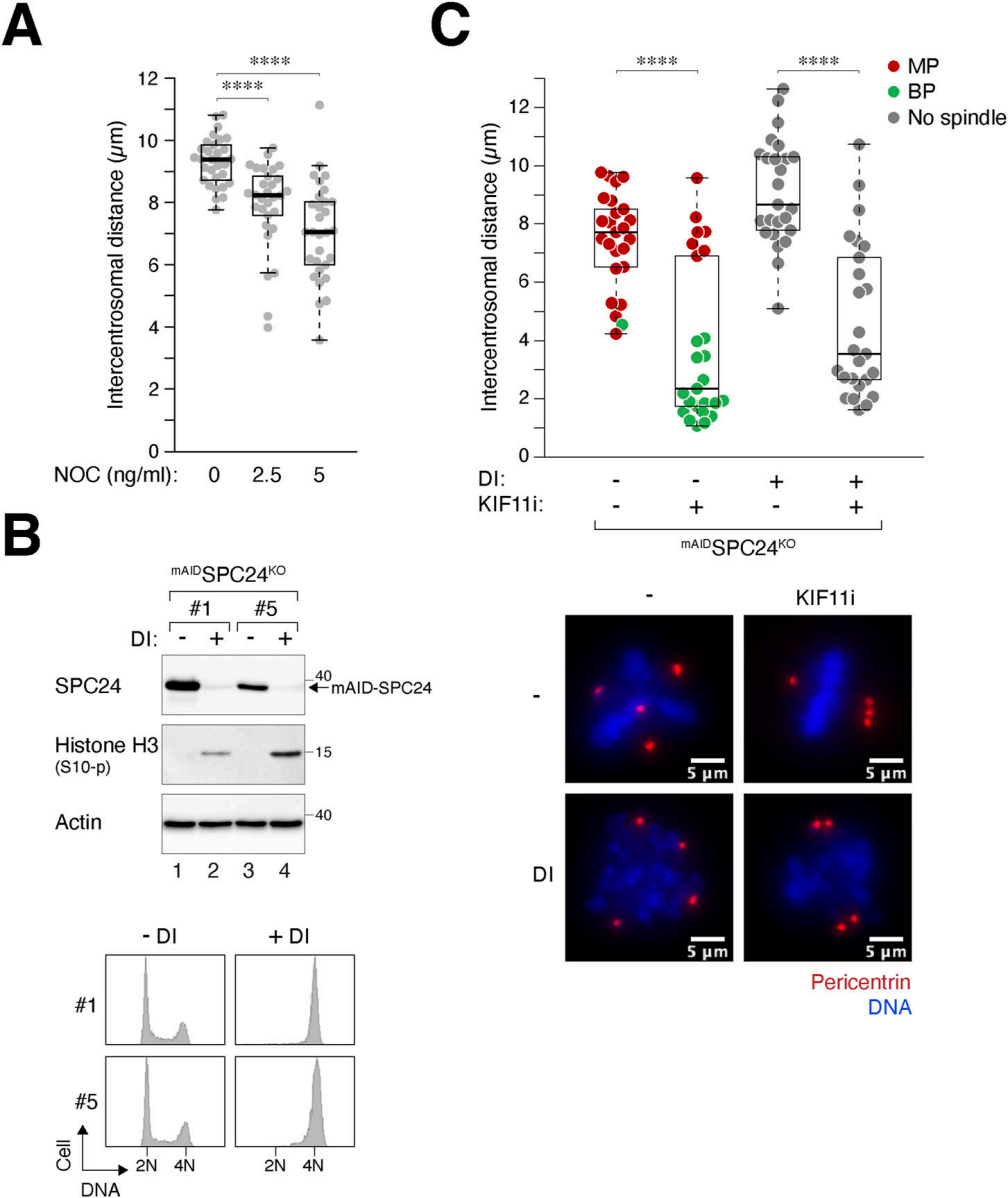
KT-MT attachment is not required for centrosome clustering after whole-genome duplication (WGD). **(A)** Partial depletion of microtubules suppresses supernumerary centrosome separation. WGD was induced in synchronized HeLa cells (Fig 1A). The cells were then treated with buffer or varying concentrations of nocodazole. Upon entry into mitosis (∼15 h), the cells were fixed and stained with antibodies against pericentrin and Hoechst 33258 for visualizing the centrosomes and DNA, respectively. Intercentrosomal distances were determined according to the method described in Fig 3E (n = 30; Mann–Whitney test, ****P < 0.0001). **(B)** Conditional silencing of SPC24. HeLa cells stably expressing mAID-SPC24 were generated. The endogenous *SPC24* was at the same time disrupted with CRISPR-Cas9. The mAID-SPC24 was resistant to the CRISPR-Cas9 because of the introduction of silent mutations at the CRISPR-Cas9 targeting site. The ^mAID^SPC24^KO^ cells were cultured in the presence of Dox and IAA (DI) for 15 h to turn off mAID-SPC24. Lysates were prepared and analyzed with immunoblotting (upper panel). The cells were also fixed and analyzed with flow cytometry (lower panel). Results from two different single-colony–derived ^mAID^SPC24^KO^ clones are shown. **(C)** Spindle attachment is dispensable for centrosome clustering. WGD was induced in ^mAID^SPC24^KO^ cells (Fig 1A). The cells were untreated or treated with KIF11i (1 nM) and/or DI to turn off the mAID-SPC24. Upon entry into mitosis, the cells were fixed and stained with antibodies against pericentrin and Hoechst 33258 to visualize the centrosomes and DNA, respectively. Intercentrosomal distances were measured according to the method described in Fig 3E (box-and-whisker plots; n = 25; Mann–Whitney test, ****P < 0.0001). Representative examples of microscopy images of declustered and clustered centrosomes are shown. Red: pericentrin; blue: DNA. Scale bar = 5 μm. Note that spindle formation was disrupted in the absence of SPC24.

Previous studies have implicated tubulin as a regulator of centrosome clustering (Kwon et al, 2008; Leber et al, 2010). To investigate the involvement of microtubules in the separation of centrosomes after WGD in MP cells, we treated cells with low concentrations of NOC to disrupt microtubule dynamics. Our findings revealed a reduction in the intercentrosomal distance during mitosis after NOC treatment, suggesting that the formation of multipolar spindles in tetraploids involves microtubule-dependent outward pushing forces (Fig 6A).

One possible interpretation of these results is that centrosome clustering may be regulated by the attachment of centrosomes to chromosomes via microtubules, rather than microtubules per se. To investigate whether the KIF11-regulated centrosome clustering depends on chromosome attachment, we disrupted the NDC80 complex, which plays a pivotal role in mediating the kinetochore–microtubule (KT-MT) attachment (Ciferri et al, 2007). Because the NDC80 complex is essential for cell survival, we disrupted *SPC24* with CRISPR-Cas9 while introducing a mini-auxin-induced degron (mAID)–tagged SPC24 into the genome (designated as ^mAID^SPC24^KO^ cells herein). The mAID-SPC24 could be acutely silenced with doxycycline and indole-3-acetic acid, which turned off the promoter and targeted mAID for degradation, respectively (Kim et al, 2024).

Depletion of mAID-SPC24 created a SPC24-deficient environment, leading to mitotic arrest (Fig 6B) and chromosome misalignment (Fig 6C). After mitotic slippage–induced WGD, centrosome clustering could still be promoted by partial inhibition of KIF11 in SPC24-deficient cells (Fig 6C), indicating that clustering of extra centrosomes does not require KT-MT attachment.

Collectively, these results indicate that the formation of multipolar spindles after WGD involves microtubules but does not depend on KT-MT attachment.

### KIFC1, but not KIFC3, antagonizes the activity of KIF11/KIF15 to drive bipolar mitosis after WGD

**Figure 7.**
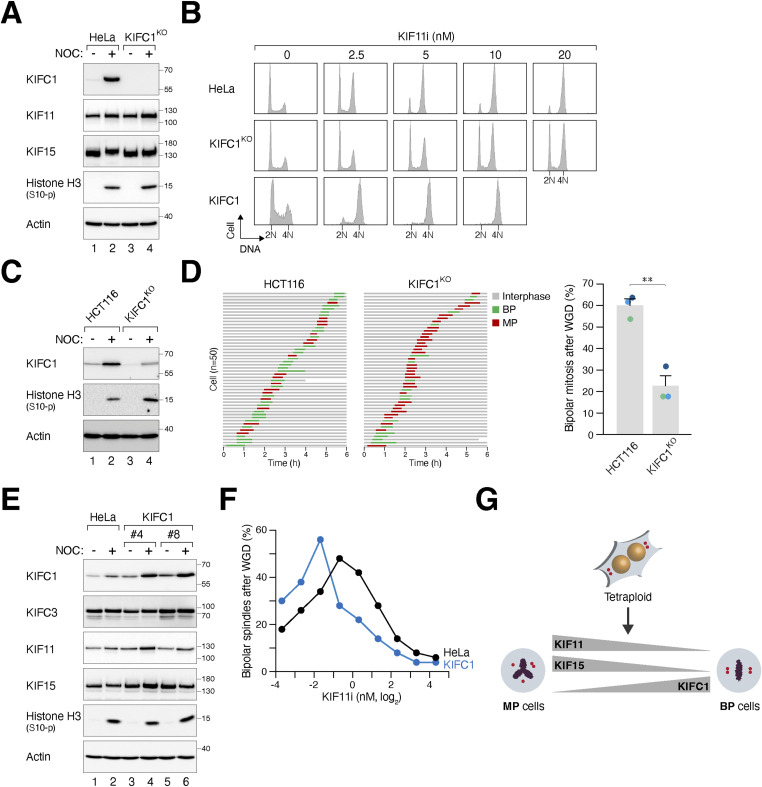
KIFC1 provides opposing forces to KIF11 in centrosome clustering after whole-genome duplication (WGD). **(A)** Silencing of KIFC1. The *KIFC1* gene was disrupted in HeLa cells using CRISPR-Cas9. Diploid HeLa and KIFC1^KO^ cells were cultured with or without nocodazole (NOC) for 18 h. Lysates were prepared and analyzed with immunoblotting. **(B)** KIFC1 regulates the sensitivity to KIF11 inhibition in diploids. Diploid HeLa, KIFC1^KO^, and KIFC1-overexpressing cells were cultured in the presence of different concentrations of KIF11i. After 18 h, cells were harvested and analyzed with flow cytometry. **(C)** Silencing of KIFC1 in HCT116 cells. The *KIFC1* gene was disrupted in HCT116 cells using CRISPR-Cas9. After selection with blasticidin for 48 h, HCT116 and KIFC1^KO^ cells (mixed population) were cultured with or without NOC for 18 h. Lysates were prepared and analyzed with immunoblotting. **(D)** Silencing of KIFC1 reduces bipolar mitosis in tetraploids. WGD was induced in HCT116 and KIFC1^KO^ cells (Fig 1A). The mitotic cell fate was tracked using live-cell imaging (n = 50). Key: interphase (gray); bipolar mitosis (green); multipolar mitosis (red); cell death (truncated bars). The percentage of cells undergoing bipolar mitosis after WGD was quantified from three independent experiments (mean ± SEM; *t* test, **P < 0.01). **(E)** Stable ectopic expression of KIFC1. HeLa cells overexpressing KIFC1 were generated (two independent clones are shown). Diploid HeLa and KIFC1-overexpressing cells were cultured with or without NOC for 18 h. Lysates were prepared and analyzed with immunoblotting. **(F)** KIFC1 overexpression promotes KIF11i-induced centrosome clustering. WGD was induced in synchronized HeLa and KIFC1-overexpressing cells (Fig 1A). The cells were treated with serial dilutions of KIF11i. The percentage of cells forming bipolar spindles was determined with immunofluorescence microscopy (n = 50). **(G)** Model of how the expression of KIF11, KIF15, and KIFC1 determines spindle polarity after WGD. After WGD, different cell lines can undergo multipolar mitosis (MP) or bipolar mitosis (BP) with clustered centrosomes. MP cells contain relatively high expression of KIF11 and KIF15, which provide outward pushing forces leading to centrosome declustering. Conversely, BP cells contain relatively low expression of KIF11 and KIF15, allowing KIFC1-dependent inward pulling forces to facilitate centrosome clustering.

**Figure S6.**
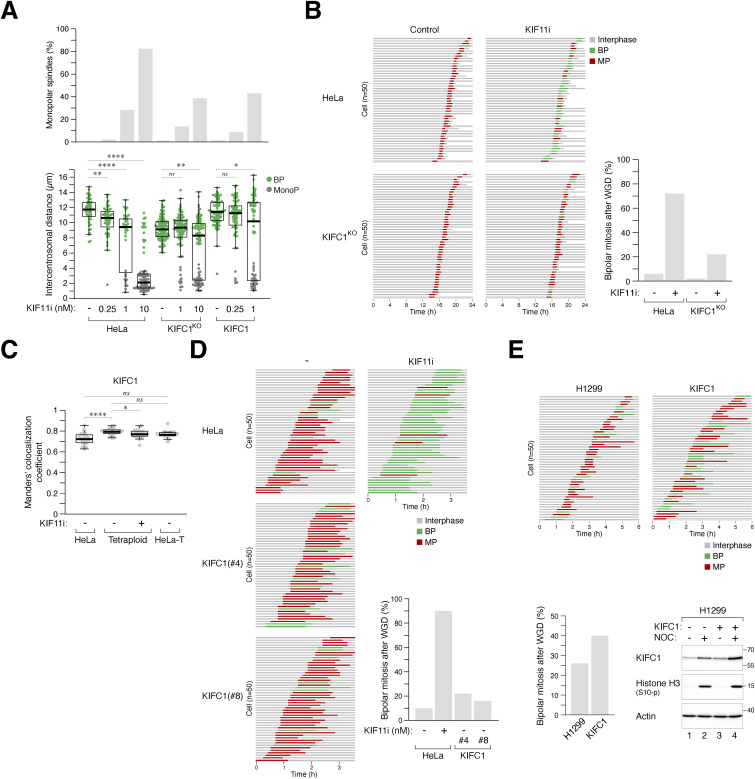
KIFC1 provides opposing forces to KIF11 in centrosome clustering after whole-genome duplication (WGD). **(A)** KIFC1 plays a role in centrosome clustering in diploids. Diploid HeLa, KIFC1^KO^, and cells ectopically expressing KIFC1 were synchronized with double thymidine block and released into medium with or without the indicated concentrations of KIF11i. Once cells entered mitosis (∼9 h), they were fixed and stained with Hoechst 33258 to visualize the DNA. The percentage of mitotic cells displaying monopolar spindles was quantified (upper panel). Intercentrosomal distances were measured by immunostaining for pericentrin (lower panel) (box-and-whisker plots; n ≥ 40; Mann–Whitney test, ****P < 0.0001; **P < 0.01; *P < 0.05; ns *P* > 0.05). **(B)** Silencing of KIFC1 counteracts KIF11i-induced bipolar mitosis in tetraploids. WGD was induced in HeLa and KIFC1^KO^ cells (Fig 1A). The cells were cultured with or without KIF11i and tracked using live-cell imaging. Key: interphase (gray); bipolar mitosis (green); multipolar mitosis (red); cell death (truncated bars). The percentage of bipolar mitosis was quantified (n = 50). **(C)** Centrosome amplification does not increase KIFC1 localization on mitotic spindles. WGD was induced in HeLa cells to generate tetraploids (Fig 1A). Diploid HeLa and tetraploid HeLa-T cells were prepared by releasing nocodazole-arrested mitotic cells into interphase. After treatment with either buffer or KIF11i for 15 h, the cells were fixed and analyzed using immunofluorescence microscopy. The localization of KIFC1 to microtubules was quantified using Manders’ colocalization coefficient (n = 20; Mann–Whitney test, ****P < 0.0001; *P < 0.05; ns *P* > 0.05). **(D)** KIFC1 overexpression marginally promotes bipolar mitosis after WGD. WGD was induced in HeLa and KIFC1-overexpressing cells (Fig 1A). The mitotic cell fate of individual cells was tracked with time-lapse microscopy. Key: interphase (gray); bipolar mitosis (green); multipolar mitosis (red); cell death (truncated bars). Tetraploid HeLa cells were treated with KIF11i as a control. The percentage of bipolar mitosis was quantified (n = 50). **(E)** KIFC1 overexpression marginally promotes bipolar mitosis in H1299 tetraploids. WGD was induced in normal and KIFC1-overexpressing H1299 (Fig 1A). The mitotic cell fate of individual cells was tracked with time-lapse microscopy. Key: interphase (gray); bipolar mitosis (green); multipolar mitosis (red); cell death (truncated bars). The percentage of bipolar mitosis was quantified (n = 50). The cells were also cultured in the presence or absence of nocodazole for 18 h. Lysates were prepared and analyzed with immunoblotting to confirm the ectopic expression of KIFC1.

KIFC1, a member of the kinesin-14 family, is of particular interest because of its strong stabilization during mitosis (Fig 7A). To assess the relative contribution of KIF11 and KIFC1 to regulating centrosome clustering after WGD, we disrupted *KIFC1* in “MP” HeLa cells using CRISPR-Cas9 (Fig 7A). Knockout of KIFC1 did not affect cell proliferation (Fig S3C), cell cycle distribution (Fig 7B), or NOC-mediated mitotic block (Fig 7A) in diploid cells. Consistent with the idea that KIFC1 generates counteracting forces to KIF11/KIF15, higher concentrations of KIF11i were required to induce G_2_/M arrest (Fig 7B) or monopolar spindle formation (Fig S6A) in KIFC1^KO^ cells compared with the parental cells. Furthermore, the percentage of bipolar mitosis induced by KIF11i after WGD was diminished in KIFC1-deficient cells (Fig S6B). Similarly, in “BP” HCT116 cells, down-regulation of KIFC1 prevented centrosome clustering and bipolar mitosis after WGD (Fig 7C and D). However, unlike KIF11 (Figs 3D and S3F), the localization of KIFC1 on microtubules did not significantly change in tetraploid cells containing extra centrosomes (Fig S6C).

Conversely, we performed experiments using KIFC1-overexpressing cells. Clones of HeLa with approximately fourfold higher protein expression compared with the endogenous KIFC1 protein level were isolated (Fig 7E). The overexpression of KIFC1 did not affect the timing of mitosis or cell cycle progression in diploids (Figs 7B and S3C). However, KIFC1 overexpression increased the sensitivity to KIF11i (Figs 7B and S6A). After WGD, KIFC1 overexpression alone slightly increased the percentage of bipolar mitosis (Fig S6D). Consequently, higher percentages of bipolar spindle formation after WGD were induced with KIF11i after KIFC1 overexpression (Fig 7F). Similar results were obtained using KIFC1-overexpressing H1299 cells (Fig S6E).

**Figure S7.**
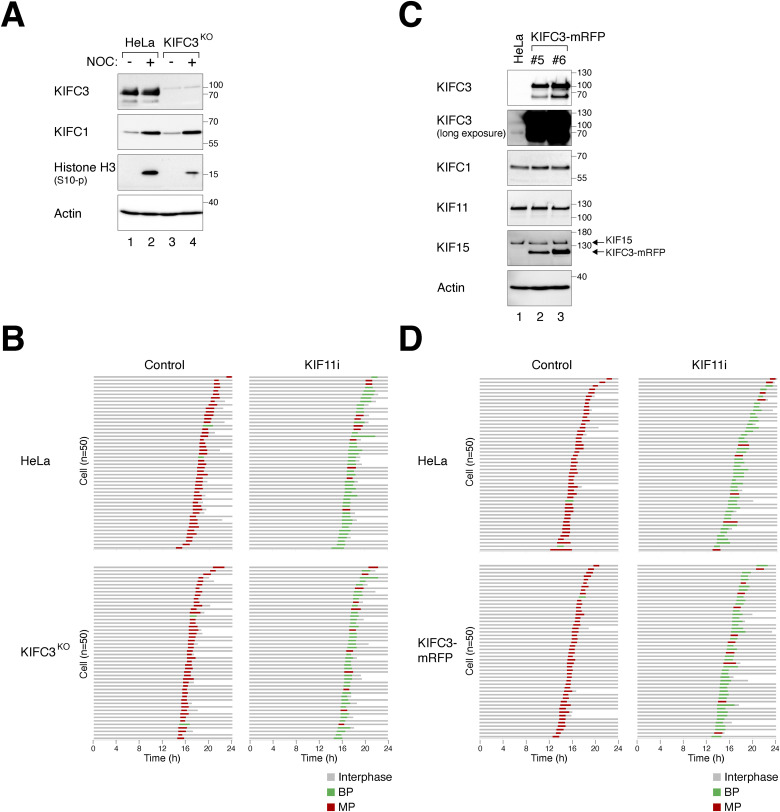
KIFC3 is not required for clustering of supernumerary centrosomes after whole-genome duplication (WGD). **(A)** Silencing of KIFC3. The *KIFC3* gene was disrupted in HeLa cells using CRISPR-Cas9. Diploid HeLa and KIFC3^KO^ cells were cultured with or without nocodazole for 18 h. Lysates were prepared and analyzed with immunoblotting. **(B)** Loss of KIFC3 does not affect KIF11i-induced bipolar mitosis after WGD. Tetraploid HeLa and KIFC3^KO^ cells generated through mitotic slippage (Fig 1A) were treated with or without KIF11i. The mitotic cell fate of individual cells was analyzed using time-lapse microscopy (n = 50). Key: interphase (gray); bipolar mitosis (green); multipolar mitosis (red); cell death (truncated bars). **(C)** Ectopic expression of KIFC3. HeLa cells overexpressing KIFC3-mRFP were generated (two independent clones are shown). Lysates were prepared and analyzed with immunoblotting. **(D)** Overexpression of KIFC3 does not affect KIF11i-induced bipolar mitosis after WGD. WGD was induced in normal and HeLa cells overexpressing KIFC3-mRFP (Fig 1A). The cells were cultured with or without KIF11i. The mitotic cell fate of individual cells was tracked using time-lapse microscopy (n = 50). Key: interphase (gray); bipolar mitosis (green); multipolar mitosis (red); cell death (truncated bars).

Another member of the kinesin-14 family, KIFC3, has been implicated in preventing premature centrosome separation after linker dissolution in diploids by opposing the outward pushing forces of KIF11 (Hata et al, 2019). The knockout or overexpression of KIFC3 did not affect cell survival in diploids (Fig S7A and C). We found that the mitotic polarity after WGD was unaffected by the loss of KIFC3 (Fig S7B) or the ectopic expression of KIFC3 (Fig S7D).

Collectively, these data suggest that KIFC1, but not KIFC3, exerts opposing forces to KIF11/KIF15 in driving centrosome clustering after WGD.

## Discussion

Agents that disrupt microtubule dynamics, such as taxanes and vinca alkaloids, can induce protracted mitotic arrest, leading to mitotic slippage and subsequent WGD. Centrosome clustering after WGD is believed to be crucial in determining genome instability, as well as influencing the efficacy of antimitotic drugs (Lau & Poon, 2023). In both the current study and a previous study by Shu et al (2019), KIF11 has emerged as a dominant factor in driving outward pushing force that regulates centrosome clustering, both in diploids and after WGD. Consistent with this, inhibition of KIF11 induced monopolar spindle formation in both diploids and tetraploids (Fig 4A).

To further investigate the impact of KIF11 inhibition in cell lines characterized by a high percentage of multipolar mitosis after WGD (MP cells), we partially inhibited KIF11 using KIF11i or siRNA. These approaches promoted centrosome clustering (Fig 4B) and facilitated the formation of bipolar spindles after WGD (Figs 4C and E and S4E). In addition, we observed higher levels of KIF11 and KIF15 in MP cell lines compared with BP cell lines. Higher colocalization of KIF11 with microtubules was also observed in tetraploids undergoing multipolar mitosis compared with those undergoing bipolar mitosis in stable tetraploid HeLa-T or RPE1-T (Figs 3D and S3F). The increase in colocalization with microtubules appears to be specific to KIF11, as no change in colocalization was observed with KIF15 or KIFC1 between BP and MP cells (Figs S5C and S6C). Together with the evidence highlighting the importance of microtubules in centrosome declustering (Fig 6), it is possible that the increased KIF11 on microtubules in MP cells may contribute to the declustering of extra centrosomes after WGD (see Fig 7G for a model).

We found that using a specific concentration of KIF11i, we were able to switch multipolar mitosis after WGD in MP cells into predominantly bipolar mitosis (Figs 4C and S4E). However, as higher concentrations of KIF11i led to monopolar arrest, the range of concentrations capable of achieving bipolar mitosis was relatively narrow (Figs 4A, 5D, and 7F). Moreover, the window of KIF11i concentrations that could promote bipolar mitosis in tetraploids without significantly inducing monopolar arrest in diploids was also limited (Fig 4A). These observations suggest that targeting KIF11 alone may not be a feasible strategy for inducing centrosome clustering and specifically promoting bipolar mitosis in cells that have undergone WGD.

A fundamental question arises regarding the molecular basis underlying the high percentage of bipolar mitosis after WGD in specific cell lines (BP cells) (Fig 2A). One potential explanation for this is the observation that BP cells tend to contain lower levels of KIF11 and KIF15 compared with MP cells (Fig 2B). However, it is unlikely that there is a straightforward correlation between spindle polarity and the expression of KIF11 alone (or in combination with KIF15 and/or KIFC1), as protein expression may not directly correspond to the activity of kinesins within the cell. Nevertheless, BP cells displayed higher sensitivity to KIF11i treatment in inducing monopolar spindle formation (Fig 2C and D), suggesting that the relatively lower expression of KIF11 reflects reduced KIF11 activity in these cells.

Similar to its role in diploid cells (Tanenbaum et al, 2009), KIF15 also contributes to the regulation of centrosome declustering after WGD, working in conjunction with KIF11. Disrupting KIF15 in diploid HeLa cells caused a slight reduction in the intercentrosomal distance but did not significantly affect cell cycle progression or bipolar spindle formation (Fig 5B and C). However, the absence of KIF15 increased the sensitivity to KIF11i, promoting the formation of monopolar spindles (Fig 5B and C). Interestingly, in MP cells such as HeLa and H1299, the loss of KIF15 led to a ∼20% increase in bipolar mitosis after WGD (Figs 5E and S5B). In fact, the presence of KIF15 alone was sufficient to maintain the frequency of multipolar mitosis in H1299 cells within our definition of “MP” cells (Fig S5B). As expected, KIF11i-induced bipolar spindle formation in MP cells was further enhanced in the absence of KIF15 (Fig 5D and E). Because silencing KIF15 did not significantly affect proliferation in diploids, targeting KIF15 could be a useful approach to induce centrosome declustering specifically in cells that have undergone WGD.

Given the high prevalence of WGD and centrosome amplification in cancer, there is considerable interest in developing therapeutic strategies that induce the declustering of extra centrosomes (Sabat-Pośpiech et al, 2019). The rationale behind these approaches is to specifically trigger detrimental multipolar mitosis in cancer cells that possess supernumerary centrosomes while sparing normal diploid cells. In this context, KIF11 is not an ideal target because of its essential role both in diploids and after WGD (Fig 4A). On the contrary, KIFC1 has been implicated in the pathogenesis of various cancer types (Pannu et al, 2015; Li et al, 2018; Han et al, 2019). Inhibiting KIFC1 has been demonstrated to preferentially sensitize cancer cells through centrosome declustering (Watts et al, 2013; Wu et al, 2013; Choe et al, 2018).

As KIFC1 is degraded by APC/C (Singh et al, 2014), the inhibition of APC/C by the spindle–assembly checkpoint resulted in the stabilization of KIFC1 (Fig 7A). It has been reported that KIF11 is also regulated by APC/C (Drosopoulos et al, 2014). However, our findings indicate that the accumulation of KIF11 during mitotic arrest is less pronounced compared with KIFC1 (Fig 7A). This suggests that the increased level of KIFC1 during mitosis may counteract the outward pushing forces exerted by KIF11 and KIF15, thereby promoting centrosome clustering (Basto et al, 2008; Kwon et al, 2008; Kleylein-Sohn et al, 2012; Chavali et al, 2016). Supporting the role of KIFC1 in centrosome clustering, silencing of KIFC1 in “BP” HCT116 cells resulted in a reduction in the frequency of bipolar mitosis after WGD (Fig 7D). Furthermore, silencing of KIFC1 in “MP” HeLa cells diminished the effectiveness of KIF11i in promoting bipolar mitosis after WGD (Fig S6B). Although the expression of KIFC1 did not significantly differ between MP and BP cell lines (Fig 2B), KIFC1 can serve as a target to promote KIF11/KIF15-dependent centrosome declustering in BP cancer cells.

The findings regarding KIFC1 contrast with that of KIFC3, another member of the kinesin-14 family that has been implicated in antagonizing KIF11’s functions in diploids (Hata et al, 2019). Unlike KIFC1, the expression of KIFC3 was not stabilized during mitosis (Fig S7A). Silencing of KIFC3 did not affect KIF11i-induced centrosome clustering after WGD in “MP” HeLa cells (Fig S7B). Whether KIFC3 plays a role in centrosome clustering in “BP” cell lines remains to be determined. Results obtained from the overexpression of KIFC1 or KIFC3 are more difficult to interpret because we do not know the activities of these recombinant proteins in the cell. Nonetheless, unlike the overexpression of KIFC3 (Fig S7D), the overexpression of KIFC1 was able to promote bipolar mitosis in “MP” cells, including HeLa and H1299 (Figs 7E and S6D and E).

Although the present study suggests that KIF11, KIF15, and KIFC1 play a pivotal role in regulating centrosome clustering after WGD in MP and BP cells, other kinesins are also likely to contribute to this process in different cell lines. In this connection, several kinesins have been implicated in regulating centrosome clustering after WGD, including KIF2C (Goupil et al, 2020), KIF18A (Cohen-Sharir et al, 2021; Marquis et al, 2021), KIF20A (Xie et al, 2022), and KIF24 (Mashima et al, 2022). However, it remains to be determined whether these and other kinesins participate in determining centrosome separation and spindle polarity after WGD in specific cell types. Further studies will be needed to fully understand the contributions of these kinesins and their interplay in centrosome dynamics after WGD.

## Materials and Methods

### Plasmids

Sleeping Beauty (SB) transposase pCMV(CAT)T7-SB100 was a gift from Zsuzsanna Izsvak (plasmid #34879; Addgene). Histone H2B-GFP in pEF/Bsd was a gift from Tim Hunt (Cancer Research UK). CRISPR-Cas9 plasmids were generated by ligating the annealing products with the indicated pairs of oligonucleotides into BbsI-cut pX330 (a gift from Feng Zhang; plasmid #42230; Addgene): KIF15 (5′-CACCGTGATCTACTGGACTCTGCAT-3′ and 5′-AAACATGCAGAGTCCAGTAGATCAC-3′); KIFC1 (5′-CACCGTTTCCAAGAAGACAGGACCC-3′ and 5′-AAACGGGTCCTGTCTTCTTGGAAAC-3′); KIFC3 (5′-CACCGCTCACACCAGCTGACCGCG-3′ and 5′-AAACCGCGGTCAGCTGGTGTGAGC-3′).

KIFC1 cDNA was prepared with a double-PCR procedure from a HeLa cDNA library (first PCRs: 5′-TTCTCTTCCACTGCATTCCC-3′ and 5′-CTGGTTCTCTTGGTCCAACG-3′; 5′-AAACGGTGCCGTGAGAGGAC-3′ and 5′-GAAACAGGAGGAGGCCAGGG-3′; 5′-CCGTGTATTCTGCCGGGTCC-3′ and 5′-AGGGACATATCGAGCATTGG-3′; and 5′-GTGTGAGATTCGCCGTGCAG-3′ and 5′-AGGGACATATCGAGCATTGG-3′; the four PCR products were then amplified in a second PCR using the first and last primers). The KIFC1 PCR product (5′-GATAAAACCGCGCTAGCCATGGATCCGCAGA-3′ and 5′-ATTGATCCCCAAGCTACAACCACCCACGGG-3′; template: KIFC1 cDNA) was inserted into NcoI- and HindIII-cut pSBbi-TIR1/Bla (Lau et al, 2021) using the Seamless Ligation Cloning Extract (SLiCE) cloning method (Motohashi, 2015) to generate a KIFC1-expressing plasmid.

KIFC3 cDNA was prepared with a double-PCR procedure from a HeLa cDNA library (first PCRs: 5′-CACCGACTTGGAGAAGCACC-3′ and 5′-CTCCTCCAGCATCTGCCCAT-3′; 5′-TGTACGAGTCAGAGCTGGAG-3′ and 5′-CTGTGGGGAGAAGACCTTGT-3′; 5′-AGCTGCGTAAGAAGTGCCACAATGA-3′ and 5′-GCTGTGCTCGTTCAGGTTGGTG-3′; and 5′-CAACAAGGTGTTTGAGTTTGGC-3′ and 5′-ATCCGTCACACAGGCAGTGG-3′; the four PCR products were then amplified in a second PCR using the first and last primers). The KIFC3-expressing plasmid was generated by inserting the PCR product (primers: 5′-CTGGCCTCTGAGGCCACCATGGTGGAGAA-3′ and 5′-TCGGATCCGTCGACTCCGGCCGAGGGCTGC-3′; template: KIFC3 cDNA) into NcoI-cut pSBbi-mRFP-myc/Bla (a gift from Lau Yan Ng, Hong Kong University of Science and Technology) using SLiCE cloning.

Histone H2B-Clover was put into a Sleeping Beauty cassette by inserting the PCR product (primers: 5′-AAAACTACCCCAAGCTGGCATGCCAGAGCC-3′ and 5′-AGAATTGATCCCCAAGCTTCTACTTGTACAGCTC-3′; template: pmTol2-H2B-Clover/Zeo [Yeung et al, 2023]) into NcoI- and HindIII-cut pSBbi-TIR1/Pur (Yeung et al, 2021) using SLiCE cloning to generate pSBbi-H2B-Clover/Pur.

### siRNAs

Stealth siRNA targeting KIF11 (GAGAGAUUCUGUGCUUUGGAGGAAA) and control siRNA were obtained from Thermo Fisher Scientific. Transfection of siRNA (40 pM) was performed using Lipofectamine RNAiMAX (Thermo Fisher Scientific) according to the manufacturer’s instructions.

### Cell culture

Cells were propagated in DMEM supplemented with 10% (vol/vol) heat-inactivated calf serum (for HeLa) or FBS (for other cell lines; 15% for U2OS and Saos-2) and 50 U/ml of penicillin–streptomycin (Thermo Fisher Scientific). RPE1 p53^KO^ cells were propagated in DMEM/F12 supplemented with 10% (vol/vol) FBS, 50 U/ml of penicillin–streptomycin, and 10 μg/ml of hygromycin B. Cells were cultured in humidified incubators at 37°C with 5% CO_2_. Synchronization procedures using double thymidine block, RO3306, and NOC shake-off were performed as previously described (Ma & Poon, 2017). Unless stated otherwise, cells were treated with the following reagents at the indicated final concentrations: blasticidin (3.75 μg/ml; Thermo Fisher Scientific), DCB (4 μM; Sigma-Aldrich), doxycycline (Dox) (2 μg/ml; Sigma-Aldrich), indole-3-acetic acid (IAA) (50 μg/ml; Sigma-Aldrich), LY2523355 (2 nM; MedChemExpress), NOC (100 ng/ml; Sigma-Aldrich), puromycin (0.3 μg/ml; Sigma-Aldrich), RO3306 (10 μM; APExBIO), SB743921 (1 nM; Selleck Chemicals), SiR-DNA (1 μM; Cytoskeleton), thymidine (2 mM; Thermo Fisher Scientific), and Z-VAD-FMK (10 μM; Enzo Life Sciences).

### Generation of stable cell lines

HeLa (cervical carcinoma) used in this study was a clone expressing the tTA (tetracycline transactivator) (Yam et al, 2000). The following cell lines were obtained from the indicated sources: H1299, HT29, MDA-MB-231, Phoenix-GP, hTERT-immortalized RPE1, Saos-2, and U2OS (American Type Culture Collection); MCF7 (a gift from Yong Xie, Hong Kong University of Science and Technology); HCT116 and HCT116 (p53^−/−^) (gifts from Bert Vogelstein, The Johns Hopkins University); Hep3B and PLC/PRF/5 (gifts from Nathalie Wong, Chinese University of Hong Kong); and Huh7 (a gift from Irene Ng, University of Hong Kong). A ^mAID^SPC24^KO^ cell line was generated as previously described (Kim et al, 2024). The p53 genes in RPE1 were disrupted using CRISPR-Cas9 (a gift from Joyce PY Mak, Hong Kong University of Science and Technology).

HeLa and H1299 cells were transfected using a calcium phosphate precipitation method (Kingston et al, 2003). Hep3, Huh7, and PLC/PRF/5 cells were transfected using Lipofectamine 3000 transfection reagent (Thermo Fisher Scientific). HCT116 cells were transfected using PolyJet transfection reagent (SignaGen Laboratories).

Knockout cell lines of KIF15, KIFC1, and KIFC3 were generated by transfecting HeLa cells with the corresponding CRISPR-Cas9 plasmids and a plasmid expressing the blasticidin-resistant gene. Transfected cells were enriched by culturing in blasticidin-containing medium for 48 h. The cells were then seeded onto 10-cm plates (for isolation of mixed population) or 96-well plates (for isolation of single-cell–derived colonies).

HeLa and H1299 overexpressing KIFC1 were generated by transfecting cells with a mixture of plasmids expressing KIFC1 (pSBbi-KIFC1/Bla) and SB transposase. After recovery for 48 h, transfected cells were selected with blasticidin for 2 wk. HeLa overexpressing KIFC3 were generated with a similar procedure except that a KIFC3-expressing construct (pSBbi-KIFC3-mRFP-myc/Bla) was used. Single-cell–derived colonies were isolated using limited dilution in 96-well plates.

HeLa and Huh7 cells stably expressing histone H2B-GFP were generated by lentiviral infection (viruses were generated in Phoenix-GP cells by cotransfection of histone H2B-GFP in pLB with VSV-G [a gift from George Tsao, the University of Hong Kong] and Ampl in pCL [a gift from Wai Jiang, The Salk Institute, USA] [Pear et al, 1993]) in the presence of 5 μg/ml of polybrene (Sigma-Aldrich). Infected cells were enriched by sorting of GFP-positive cells using flow cytometry. Hep3B cells stably expressing histone H2B-GFP were generated by transfecting cells with histone H2B-GFP in pEF/Bsd. After selection in blasticidin-containing medium for 2 wk, single-cell–derived colonies were isolated using cloning cylinders. PLC/PRF/5 cells stably expressing histone H2B-Clover were generated by transfecting cells with a mixture of plasmids expressing pSBbi-H2B-Clover/Pur and SB transposase. Histone H2B-Clover–positive cells were enriched by propagating in medium supplemented with puromycin for 2 wk.

Down-regulation of KIF15 and KIFC1 in H1299 and HCT116 cells, respectively, was achieved by cotransfection using a mixture of the corresponding CRISPR-Cas9 plasmids, pSBbi-H2B-Clover/Pur, and SB transposase. H1299 cells overexpressing KIFC1 were generated by transfecting cells with pSBbi-KIFC1/Bla, pSBbi-H2B-Clover/Pur, and SB transposase. Transfected cells were enriched by propagating in medium supplemented with puromycin (and blasticidin for KIFC1 overexpression) for 2 wk.

### Generation of tetraploids

For inducing mitotic slippage, cells were synchronized with a double thymidine block procedure and released into NOC-containing medium (Ma & Poon, 2017). Mitotic cells were collected by mechanical shake-off at 12 h after thymidine release and then forced to exit mitosis by treating with RO3306 for 2 h. After washing twice with PBS to remove unattached cells and chemical inhibitors, fresh medium was added for further incubation. Cytokinesis failure was induced by propagating cells in medium supplemented with DCB for 18 h. When coupled with synchronization, cells were first synchronized with RO3306 for 15 h. Cells were then washed twice with PBS and released into fresh medium supplemented with DCB for 5 h. The cells were washed twice with PBS again and further incubated in fresh medium.

To establish stable tetraploid cell lines HeLa-T and RPE1-T, mitotic slippage was induced as described above. For HeLa, the cells were then cultured in medium containing KIF11i (1 nM) for 48 h before being seeded onto 96-well plates for isolation of single-cell–derived colonies. For RPE1, single cells containing high forward scatter (FSC) were sorted using flow cytometry and seeded onto 96-well plates. The ploidy and the number of spindle poles of individual single colonies were examined by flow cytometry and immunofluorescence microscopy, respectively.

### Flow cytometry

Flow cytometry analysis after propidium iodide staining was performed as previously described (Mak et al, 2020). In brief, cells were trypsinized, washed with PBS, and fixed with ice-cold 80% ethanol for 30 min. The cells were then stained with a solution containing 40 μg/ml of propidium iodide and 40 μg/ml of RNase A at 37°C for 30 min. The DNA content of 10,000 cells was analyzed using a FACSAria III flow cytometer (BD Biosciences).

### Live-cell imaging

Cells stably expressing histone H2B-GFP or histone H2B-Clover were seeded onto 12- or 24-well plates and placed into an automated microscopy system equipped with temperature, humidity, and CO_2_ control chamber (Zeiss Celldiscoverer 7). For other cell lines, nuclei were labeled with a live-cell DNA dye SiR-DNA, added 1 h before the start of imaging. Images were captured at 5- or 10-min intervals for a duration of up to 40 h. Data acquisition was performed using a 5X/0.35 Plan Apo dry objective with 2X afocal magnification and Zeiss ZEN 2.3 (blue edition). Subsequently, image analysis was conducted using ImageJ (National Institutes of Health). Mitosis was defined as the period from the DNA condensation to the anaphase onset. After mitosis, one of the daughter cells was randomly selected and continued to be tracked.

### Antibodies and immunological methods

The following antibodies were obtained from the indicated sources: alpha-tubulin (EP1332Y) (ab52866; Abcam), Alexa Fluor 488–conjugated alpha-tubulin (11H10) (5063S; Cell Signaling Technology), beta-actin (AC-74) (A5316; Sigma-Aldrich), centrin-1 (20H5) (04-1624; Sigma-Aldrich), phosphorylated histone H3^Ser10^ (sc-8656R; Santa Cruz Biotechnology), KIFC1 (12313S; Cell Signaling Technology), KIFC3 (D-9) (sc-365494; Santa Cruz Biotechnology), KIF11 (BD611186; BD Biosciences), KIF15 (36-1) (sc-1009489; Santa Cruz Biotechnology), pericentrin (ab220784; Abcam), and SPC24 (A16601; ABclonal Technology). Immunoblotting was performed as previously described (Ng et al, 2019). Unless stated otherwise, equal amounts of lysates (10 μg) were loaded per lane. In some experiments, lysates from a specific number of cells were loaded as indicated. The band intensities of KIF11, KIF15, and KIFC1 were quantified with Image Lab software (version 5.2.1 build 11; Bio-Rad Laboratories). The positions of molecular size standards (in kD) are indicated in the figures.

### Immunofluorescence microscopy

Samples for immunofluorescence analysis were prepared as previously described (Lau et al, 2021). In brief, cells were cultured on coverslips coated with 0.1% poly-L-lysine and fixed with ice-cold methanol at −20°C for 10 min. Samples were permeabilized with 0.4% Triton X-100 in PBS at 25°C for 30 min, followed by blocking using 0.2% BSA in PBS at 25°C for 30 min. The centrosomes and KIF11 were labeled with primary antibodies against pericentrin or centrin-1, and KIF11, respectively (added sequentially at 25°C for 1 h each). The corresponding secondary antibodies including Alexa Fluor 488 goat anti-mouse IgG, Alexa Fluor 568 goat anti-rabbit IgG, Alexa Fluor 594 goat anti-rabbit IgG, and Alexa Fluor 633 goat anti-mouse IgG (Thermo Fisher Scientific) were then added sequentially at 25°C, each for 1 h. For immunolabeling microtubules, samples were incubated with Alexa Fluor 488–conjugated alpha-tubulin antibodies at 4°C overnight. Nuclei were counterstained using 200 ng/ml of Hoechst 33258 at 25°C for 10 min. Samples were washed with 0.1% Triton X-100 in PBS three times (5 min each) between each labeling step. After the final wash, the cells were mounted onto coverslips with 2% N-propyl gallate (Sigma-Aldrich) in glycerol.

Data acquisition was carried out with a Zeiss Celldiscoverer 7 fluorescence microscope equipped with Zeiss ZEN 2.3 (blue edition). Z-stack images were captured using a 20X/0.95 Plan Apo dry objective with 2X afocal magnification or a 50X/1.2 Plan Apo water-immersion objective, covering a thickness of 12 μm (step size: 0.4 μm). Centrin-1 signals were acquired using a Leica TCS SP8 confocal microscope using a 63X/1.4 oil-immersion objective. Representative images shown are maximal projections of captured Z-stack images. Intercentrosomal distances were determined by measuring the three-dimensional separation between the center of pericentrin foci across the Z-stack images using ImageJ (National Institutes of Health). Average intensity was calculated as the sum of intensity divided by the number of pixels within an object. The localization of KIF11 to microtubules was assessed using Manders’ colocalization coefficient, calculated using the JACoP plugin in ImageJ.

### Statistical analysis

Box-and-whisker plots were generated using RStudio (version 1.2.5019; Boston) and Prism (version 9.5.1; GraphPad Software, LLC). The center lines represent the medians, the box limits indicate the interquartile range, and the whiskers extend to the most extreme data points that were no more than 1.5 times the interquartile range from the 25^th^ and 75^th^ percentiles. Statistical significance was determined using the Mann–Whitney *U* test or *t* test. The Kruskal–Wallis *H* test with post hoc Dunn–Bonferroni’s multiple comparison test was used to calculate statistical significance with more than two independent samples. Linear regression analysis was used to assess the statistical significance in the relationship between KIF11i vulnerability and frequencies of bipolar division after WGD of different cell lines.

## Supplementary Material

Reviewer comments

## Data Availability

All primary data are available upon request.
